# TDP-43, a protein central to amyotrophic lateral sclerosis, is destabilized by tankyrase-1 and -2

**DOI:** 10.1242/jcs.245811

**Published:** 2020-06-23

**Authors:** Leeanne McGurk, Olivia M. Rifai, Nancy M. Bonini

**Affiliations:** Department of Biology, University of Pennsylvania, Philadelphia, PA 19104, USA

**Keywords:** ALS, FTD, PARP, Poly(ADP-ribose), Proteasome, Ubiquitin

## Abstract

In >95% of cases of amyotrophic lateral sclerosis (ALS) and ∼45% of frontotemporal degeneration (FTD), the RNA/DNA-binding protein TDP-43 is cleared from the nucleus and abnormally accumulates in the cytoplasm of affected brain cells. Although the cellular triggers of disease pathology remain enigmatic, mounting evidence implicates the poly(ADP-ribose) polymerases (PARPs) in TDP-43 neurotoxicity. Here we show that inhibition of the PARP enzymes tankyrase 1 and tankyrase 2 (referred to as Tnks-1/2) protect primary rodent neurons from TDP-43-associated neurotoxicity. We demonstrate that Tnks-1/2 interacts with TDP-43 via a newly defined tankyrase-binding domain. Upon investigating the functional effect, we find that interaction with Tnks-1/2 inhibits the ubiquitination and proteasomal turnover of TDP-43, leading to its stabilization. We further show that proteasomal turnover of TDP-43 occurs preferentially in the nucleus; our data indicate that Tnks-1/2 stabilizes TDP-43 by promoting cytoplasmic accumulation, which sequesters the protein from nuclear proteasome degradation. Thus, Tnks-1/2 activity modulates TDP-43 and is a potential therapeutic target in diseases associated with TDP-43, such as ALS and FTD.

This article has an associated First Person interview with the first author of the paper.

## Abstract (precis)

**Highlighted Article:** TDP-43 interacts with Tnks-1/2 via a tankyrase-binding domain. This interaction promotes the cytoplasmic accumulation and stabilization of TDP-43.

## INTRODUCTION

Amyotrophic lateral sclerosis (ALS) and frontotemporal degeneration (FTD) are fatal and incurable disorders at two ends of a disease spectrum called ALS/FTD (Gao et al., 2017; Ling et al., 2013). Although the full molecular underpinnings of ALS/FTD are unclear, ∼5-10% of ALS and 25-50% of FTD cases are inherited, implicating a genetic cause (Mackenzie and Neumann, 2016; Taylor et al., 2016). Known mutations are enriched in genes with roles in RNA metabolism, autophagy and the ubiquitin proteasome system (UPS) (Balendra and Isaacs, 2018; Gao et al., 2017; Mackenzie and Neumann, 2016; Taylor et al., 2016). Furthermore, at the endpoint of >95% of ALS cases and ∼45% of FTD cases, the RNA/DNA-binding protein TDP-43 (TAR DNA-binding protein of 43 kDa) abnormally accumulates in ubiquitin-positive inclusions in the cytoplasm of affected neurons and glia (Arai et al., 2006; Mackenzie et al., 2007; Neumann et al., 2006). Many disease-associated mutations in TDP-43 have been identified in familial and sporadic ALS and occasionally in FTD, implicating TDP-43 dysfunction as central to disease (Benajiba et al., 2009; Borroni et al., 2009; Floris et al., 2015; Kabashi et al., 2008; Kovacs et al., 2009; Sreedharan et al., 2008; Synofzik et al., 2014; Van Deerlin et al., 2008). Although the ubiquitination and subsequent turnover of TDP-43 has been linked to the UPS and autophagy in cellular systems (Hans et al., 2014; Kim et al., 2009; Liu et al., 2014; Scotter et al., 2014; van Eersel et al., 2011; Winton et al., 2008; Zhang et al., 2010), if and how these degradation pathways regulate the pathobiology of TDP-43 in human disease remains unknown.

Mounting evidence implicates poly(ADP-ribose) (PAR) activity in the neurotoxicity associated with ALS/FTD and other neurodegenerative disorders such as ischemic stroke, Parkinson's and Alzheimer's diseases (Duan et al., 2019; Fatokun et al., 2014; Kam et al., 2018; McGurk et al., 2018a,b,c, 2019; Naumann et al., 2018; Rulten et al., 2014). PAR is a reversible post-translational modification generated by PAR polymerases (PARPs), whereby polymers of ADP-ribose are covalently linked onto target proteins (transPARylation) or the enzymes themselves (autoPARylation) (Gupte et al., 2017). An added layer of regulation occurs via noncovalent binding between the PAR polymer and target proteins that harbor PAR-binding domains such as the PAR-binding motif (PBM) (Teloni and Altmeyer, 2016). In mammals, there are four enzymes with PAR activity: PARP-1, PARP-2, tankyrase-1 and tankyrase-2 (Vyas et al., 2014). Of particular interest are the tankyrases, which are two closely related paralogues, collectively referred to as Tnks-1/2 (Citarelli et al., 2010). Tnks-1 and Tnks-2 both have a PARP domain, a sterile alpha motif (SAM) and five ankyrin-repeat clusters (ARCs) (Citarelli et al., 2010). The SAM domain mediates self-polymerization of Tnks-1/2, which is a biophysical property required for Tnks-1/2 function (De Rycker and Price, 2004; De Rycker et al., 2003; Mariotti et al., 2016).The ARCs bind to a loosely conserved tankyrase-binding motif (TBM) known as RxxΦDG (where x represents any amino acid and Φ is a small hydrophobic amino acid or glycine) that is embedded in target proteins (DaRosa et al., 2018; Guettler et al., 2011; Morrone et al., 2012; Sbodio and Chi, 2002). A central role of Tnks-1/2 is in protein turnover by the UPS (Bhardwaj et al., 2017; Chang et al., 2003; Cho-Park and Steller, 2013; Huang et al., 2009; Levaot et al., 2011; Li et al., 2017b; Zhang et al., 2011).

The first known protein substrate of Tnks-1/2 was the telomere-binding protein TRF1 (Rippmann et al., 2002; Smith et al., 1998). PARylation of TRF-1 releases TRF-1 from DNA, resulting in telomere elongation (Cook et al., 2002; Smith and de Lange, 2000). PARylated TRF-1 released from telomeres is subsequently ubiquitinated and degraded (Chang et al., 2003). Further involvement of Tnks-1/2 in ubiquitin-mediated proteolysis was shown by studies of axin in the Wnt-signaling pathway (Callow et al., 2011; Croy et al., 2016; Feng et al., 2014; Huang et al., 2009; Zhang et al., 2011). Under basal conditions, Tnks-1/2 PARylates axin, then the PAR polymer recruits an E3-ubiquitin ligase that subsequently ubiquitinates axin, and this directs axin to the proteasome (Callow et al., 2011; Croy et al., 2016; Feng et al., 2014; Huang et al., 2009; Zhang et al., 2011). However, upon Wnt stimulation, PARylation of axin stabilizes axin at the Wnt receptor (Wang et al., 2016; Yang et al., 2016). Tnks-1/2 is known to regulate proteasomal turnover of several other proteins including APC2, 3BP2, HIPPO, PTEN, TRF1 and YAP1 (Bhardwaj et al., 2017; Callow et al., 2011; Chang et al., 2003; Croy et al., 2016; Levaot et al., 2011; Li et al., 2015). The role of Tnks-1/2 and PAR-dependent protein degradation pathways in neurodegenerative diseases such as ALS/FTD is still largely unexplored.

Recently, we discovered in *Drosophila melanogaster* that reduction of the Tnks-1/2 homologue mitigates the neurotoxicity of TDP-43, whereas upregulation exacerbates TDP-43-associated toxicity (McGurk et al., 2018a). Furthermore, we observed that downregulation of the Tnks-1/2 homologue led to an increase in nuclear TDP-43 and a decrease in cytoplasmic TDP-43 in *Drosophila* neurons (McGurk et al., 2018a). Given the role of Tnks-1/2 in protein degradation and the role of aberrant protein degradation in ALS/FTD, we sought to determine whether Tnks-1/2 promotes ubiquitination and degradation of TDP-43. We demonstrate that a highly selective inhibitor of Tnks-1/2 activity mitigates the neurotoxicity of TDP-43 to rodent neurons. We discovered that TDP-43 has a functional tankyrase-binding motif; however, our data show that TDP-43 is not degraded by Tnks-1/2-dependent ubiquitination. By contrast, our results suggest that Tnks-1/2 stabilizes TDP-43 and that this may occur by inhibiting degradation of TDP-43 by the nuclear proteasome. These findings provide molecular and cellular insight into the interaction between Tnks-1/2 and TDP-43 and provide a foundation for developing novel therapeutic strategies for TDP-43-associated diseases.

## RESULTS

### Pharmacological inhibition of tankyrase-1/2 protects against TDP-43-associated toxicity in rodent primary neurons

**Fig. 1.**
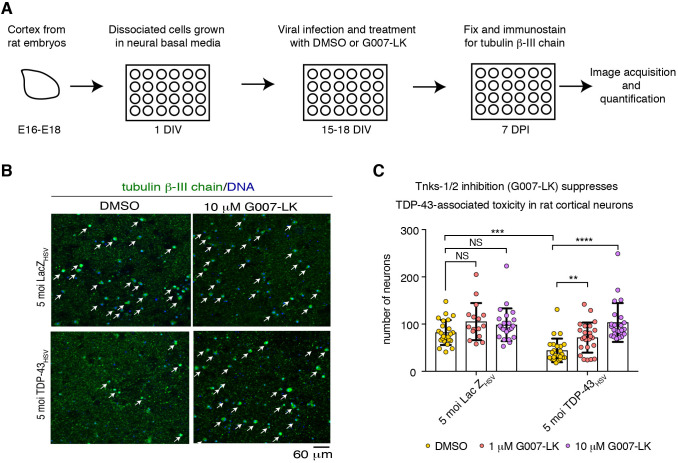
**Tnks-1/2 inhibitor G007-LK reduces TDP-43-associated loss of rat primary cortical neurons.** (A) Cortical neurons isolated from Sprague Dawley embryos (E16-E18) were seeded in 24-well plate format at a density of 100,000 neurons. After 15-18 days *in vitro* (DIV) neurons were virally infected with either HSV-LacZ or HSV-TDP-43 and treated with DMSO or G007-LK. Neurons were fixed and immunostained at 7 days post infection (DPI). See Fig. S2 for expanded images. (B) Neurons were immunolabeled for the neuronal marker tubulin β-III chain (green) and counterstained with Hoechst 33342 (blue). Arrows indicate neurons. (C) Viral infection with HSV-TDP-43 at 5 moi resulted in significant loss in cortical neurons compared with the HSV-LacZ control. Co-treatment with G007-LK (at 1 and 10 µM) significantly suppressed TDP-43-associated neuronal loss. Graph shows individual data points and the mean±s.d. from the same pregnant female analyzed by one-way ANOVA (*P*<0.0001) and Dunnett's test. See Fig. S1B,C for two other independent biological repeats. NS, not significant; **P<0.01, ***P<0.001, ****P<0.0001.

Our previous studies demonstrate that Tnks is a dose-sensitive modifier of TDP-43-associated toxicity in *Drosophila*: upregulation of Tnks enhances TDP-43-associated toxicity whereas downregulation of Tnks mitigates TDP-43-associated toxicity (McGurk et al., 2018a). To ascertain whether Tnks-1/2 inhibition is of therapeutic benefit in mammalian cells, we developed a TDP-43 neurotoxicity assay in rat primary cortical neurons (Fig. 1A; Fig. S1A). Primary neurons were virally infected with 5 multiplicity of infection (moi) of an attenuated herpes simplex virus encoding either a control protein (LacZ) or human TDP-43. Cultures were maintained for 7 days post infection, then immunostained for the neuronal marker Tubulin β-III chain. Surviving neuronal cell bodies were quantified. TDP-43 expression resulted in a significant reduction in the number of cortical neurons compared with the LacZ control [44±25 versus 82±26 (s.d.), respectively] (Fig. 1B,C; Fig. S1B,C, Fig. S2), indicating that virally induced expression of TDP-43 leads to neuronal toxicity in rat primary cortical neurons.

To determine whether Tnks-1/2 inhibition could protect neurons from TDP-43-associated toxicity, we examined the effect of the small-molecule inhibitor G007-LK, as it is highly selective for Tnks-1/2 and has no reported effect on PARP-1 activity (Voronkov et al., 2013). To determine whether treatment with G007-LK had any effect on neurons in the absence of TDP-43-associated toxicity, we treated cultured neurons infected with HSV-LacZ with either vehicle (DMSO) or G007-LK (1 or 10 µM) for 7 days and quantified the number of neuronal cell bodies immunolabeled with Tubulin β-III chain. At the concentrations tested, G007-LK treatment had no significant effect on neuronal number (Fig. 1B,C; Fig. S1B,C, Fig. S2), indicating that G007-LK had little to no effect on general neuronal survival. By contrast, neuronal loss induced by HSV-TDP-43 was significantly reduced by treatment with 1 or 10 µM G007-LK (Fig. 1B,C; Fig. S1B,C, Fig. S2), indicating that G007-LK protects neurons from TDP-43-associated toxicity. These data demonstrate that treating rat primary cortical neurons with the Tnks-1/2 inhibitor protects against TDP-43-associated toxicity. Furthermore, they suggest that targeting Tnks-1/2 activity in TDP-43-associated disease is a potential therapeutic strategy.

### TDP-43 harbors a functional tankyrase-binding motif

**Fig. 2.**
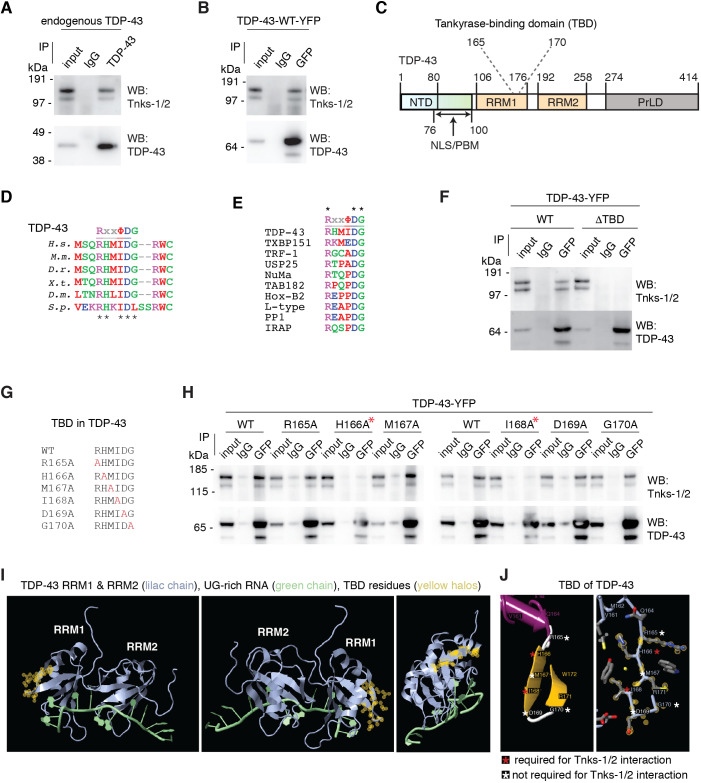
**TDP-43 and tankyrase-1/2 interact via a tankyrase-binding domain.** (A) Endogenous Tnks-1/2 co-immunoprecipitated with endogenous TDP-43 in COS-7 cells. Experiment repeated three independent times. (B) Endogenous Tnks-1/2 co-immunoprecipitated with human TDP-43-WT-YFP in COS-7 cells. Experiment repeated four independent times. (C) TDP-43 conserved protein domains. The putative tankyrase-binding domain is amino acids 165-170. NTD, N-terminal domain; NLS, nuclear localization sequence; PBM, PAR-binding motif; RRM, RNA recognition motif; PrLD, prion-like domain. (D) TDP-43 amino acids 165-170 are evolutionarily conserved among *Homo sapiens* (*H.s.*), *Mus musculus* (*M.m*), *Danio rerio* (*D.r.*), *Xenopus tropicalis* (*X.t.*)*, Drosophila melanogaster* (*D.m.*) and *Stronglyocentrotus purpuratus* (*S.p.*)*.* Black asterisks indicate the evolutionarily conserved amino acids. The aspartic acid at position 169 is mutated to glycine (D169G) in a case of sporadic ALS (Kabashi et al., 2008). (E) Alignment of TDP-43 amino acids 165-170 to tankyrase-binding domains in Tax1-binding protein 1 (TXBP151), telomere-repeat binding factor 1 (TRF-1), ubiquitin-specific peptidase 25 (USP25), nuclear mitotic apparatus protein (NuMA), 182 kDa tankyrase-binding protein (TAB182), homeobox B2 (Hox-B2), L-type calcium channel (L-type), protein phosphatase 1 (PP1) and insulin-responsive aminopeptidase (IRAP) (Sbodio and Chi, 2002). Asterisks indicate conserved amino acids in this set of proteins. (F) Deletion of the predicted TBD in TDP-43 prevents endogenous Tnks-1/2 co-immunoprecipitating with human TDP-43-WT-YFP in COS-7 cells. Experiment was repeated six independent times. (G) A series of TDP-43-YFP constructs were generated with the amino acids in the TBD mutated to alanine (highlighted in red). (H) Mutation of either H166 or I168 to alanine (red asterisks) was sufficient to abolish the interaction between endogenous Tnks-1/2 and TDP-43-YFP in COS-7 cells, whereas mutation of R165, M167, D169 or G170 to alanine had little to no effect. Experiment was repeated three independent times. (I) The previously reported NMR structure of RRMs of TDP-43 binding to UG-rich RNA (4BS2) (Lukavsky et al., 2013). The TBD was identified using FirstGlance in Jmol. The yellow halos mark the structure of each amino acid in the TBD. (J) The amino acids (H166 and I168) in the TBD that are crucial for the interaction between Tnks-1/2 and TDP-43 are marked with a red asterisk and those that have no to little effect on the interaction with Tnks-1/2 when mutated to alanine are marked with a white asterisk.

To explore molecular interactions between TDP-43 and Tnks-1/2, including the possibility that TDP-43 and Tnks-1/2 function together in a protein complex, we established a robust series of co-immunoprecipitation assays in mammalian cells. We consistently observed that both endogenous TDP-43 and an exogenously expressed wild-type (WT) form of TDP-43 (TDP-43-WT-YFP) co-immunoprecipitate with endogenous Tnks-1/2 in COS-7 cells and HEK-293T cells (Fig. 2A,B; Fig. S3A,B). These data demonstrate that TDP-43 and Tnks-1/2 interact in mammalian protein lysates.

To determine whether there is a region within TDP-43 that may directly interact with Tnks-1/2, we computationally aligned the tankyrase-binding motif (RxxΦDG) to the human TDP-43 protein sequence. This highlighted an evolutionarily conserved region (amino acids 165-170) with 84% identity to the tankyrase-binding motif (Fig. 2C-E; Fig. S3A), which we call the tankyrase-binding domain (TBD) (Fig. 2C). To establish whether the predicted tankyrase-binding motif in TDP-43 could mediate interaction with Tnks-1/2, we deleted the TBD from TDP-43 (TDP-43-ΔTBD-YFP) and tested the ability of the mutant protein to co-immunoprecipitate with Tnks-1/2. The results revealed that deletion of the TBD abolished the capacity of TDP-43 to co-immunoprecipitate with Tnks-1/2 in mammalian cells (Fig. 2F). Importantly, deletion of the TBD did not affect all interactions, as it had no effect on the capacity of TDP-43-ΔTBD to co-immunoprecipitate with endogenous TDP-43 from cellular lysates (Fig. S3D). Collectively, these data suggest that the TBD is essential for the interaction between TDP-43 and Tnks-1/2.

To define further the Tnks-1/2 interaction domain in TDP-43, we mutated each amino acid in the TBD (RxxΦDG) individually to alanine (Fig. 2G) and tested the ability of the mutated protein variants to co-immunoprecipitate with Tnks-1/2. This demonstrated that mutation of either H166 or I168 to alanine was sufficient to abolish the interaction between TDP-43-YFP and Tnks-1/2, whereas mutation of R165, D169 or G170 to alanine had little to no effect (Fig. 2H). We note that the ALS-associated mutation of TDP-43 D169G (Kabashi et al., 2008) resides within the TBD of TDP-43; however, similar to the alanine mutation in D169, the mutation to glycine had no effect on the co-immunoprecipitation of TDP-43 with Tnks-1/2 (Fig. S3E).

Analysis of the previously solved NMR structure of RNA recognition motifs (RRM1 and RRM2) of TDP-43 (Lukavsky et al., 2013) revealed that the TBD and the RNA-binding regions are on opposite sides of RRM1 (Fig. 2I). Recent studies have also demonstrated that mutation in the TBD region (D169G) has no effect on RNA-binding (Chen et al., 2019). The TBD spans a loop, a β-strand and a second loop (Fig. 2J) and, intriguingly, the amino acids essential for the interaction with Tnks-1/2 (H166 and I168) are positioned on the internal side of the β-strand (Fig. 2J). The nonessential amino acids of the TBD are located on the unstructured loops (R165, D169 and G170) or on the external surface of the β-strand (M167) (Fig. 2J). These combined data indicate that TDP-43 and Tnks-1/2 interact and that this interaction is dependent upon H166 and I168, which are positioned in the β-strand in the TBD of TDP-43.

### Tankyrase-1/2 inhibits ubiquitination and proteasomal turnover of TDP-43

**Fig. 3.**
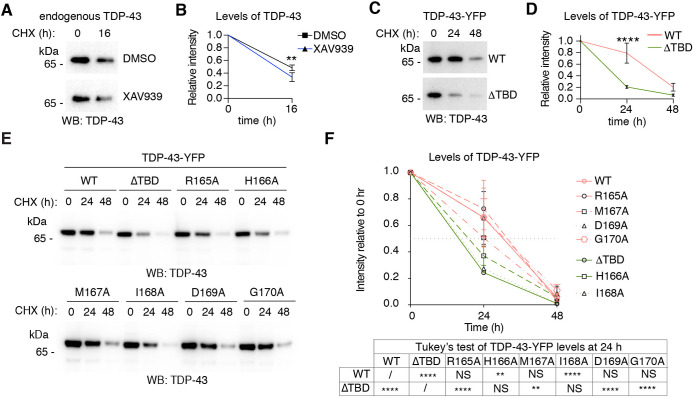
**Tnks-1/2 regulates the degradation of TDP-43.** (A) Cells were treated with the protein-synthesis inhibitor cycloheximide (CHX) in the presence of vehicle (DMSO) or the Tnks-1/2 inhibitor XAV939 (10 µM). The levels of endogenous TDP-43 were assessed by immunoblot. (B) Endogenous TDP-43 was less stable in the presence of XAV939 compared with DMSO-treated control. Mean±s.d. of three independent experiments. Two-way ANOVA (*P*<0.01) and Sidak's multiple comparison test. (C). Cells expressing TDP-43-WT-YFP and TDP-43-ΔTBD-YFP were treated with CHX, and protein levels were determined by immunoblot for TDP-43. (D) TDP-43-ΔTBD-YFP was less stable than TDP-43-WT-YFP upon CHX treatment. Mean±s.d. of three independent experiments. Two-way ANOVA (*P*<0.0001) and Sidak's multiple comparison test. (E) Cells expressing TDP-43-YFP (-WT, -ΔTBD, -R165A, -H166A, -M167A, -I168A, -D169A and -G1870A were treated with CHX and protein levels determined by immunoblot. (F) Mutations in the TBD of TDP-43-YFP that abolish the interaction with Tnks-1/2 (ΔTBD, H166A or I168A) led to a reduction in protein stability compared with TDP-43-WT. By contrast, the stability of TDP-43 with mutations in the TBD that do not affect the interaction with Tnks-1/2 (R165A, M167A, D169A or G170A) was no different from the stability of TDP-43-WT. NS, not significant; **P<0.01, ****P<0.0001.

The ADP-ribosylation activity of Tnks-1/2 has been established to promote proteasomal turnover of Tnks-1/2 substrates including APC2, axin, 3BP2, HIPPO, PTEN, TRF1 and YAP1 (Bhardwaj et al., 2017; Callow et al., 2011; Chang et al., 2003; Croy et al., 2016; Huang et al., 2009; Levaot et al., 2011; Li et al., 2015). To test the possibility that Tnks-1/2 regulates the levels of TDP-43, we treated cells with the protein-synthesis inhibitor cycloheximide and measured the degradation of endogenous TDP-43 in the presence of vehicle (DMSO) or the Tnks-1/2 inhibitor XAV939. Surprisingly, treatment with XAV939 led to a slight but significant decrease (*P*<0.01) in the levels of endogenous TDP-43 protein compared with the vehicle control (Fig. 3A,B). This finding indicates that inhibition of Tnks-1/2 promotes the degradation of endogenous TDP-43. To rule out potential off-target effects of the Tnks-1/2 inhibitor, we compared the degradation of TDP-43-WT-YFP with that of the forms of TDP-43-YFP unable to interact with Tnks-1/2 (TDP-43-ΔTBD, -H166A and -I168A). Consistent with the effect of the Tnks-1/2 inhibitor on endogenous TDP-43 (Fig. 3A,B), the levels of TDP-43-YFP unable to interact with Tnks-1/2 (TDP-43-ΔTBD, -H166A and -I168A) were significantly reduced compared with forms of TDP-43-YFP that interact with Tnks-1/2 (-WT, -R165A, -M167A, -D169A and -G170A) (Fig. 3C-F; Fig. S4). Together, these data indicate that loss of the Tnks-1/2 interaction leads to increased degradation of TDP-43 and suggest that under normal conditions Tnks-1/2 functions to stabilize TDP-43.

**Fig. 4.**
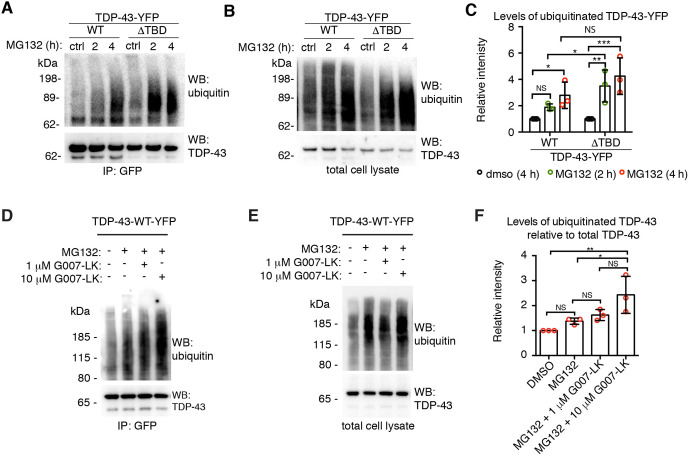
**Tnks-1/2 regulates the ubiquitination of TDP-43.** (A,B) Cells expressing TDP-43-WT-YFP or TDP-43-ΔTBD-YFP were exposed to vehicle (DMSO) or 10 µM MG123. Immunoprecipitated TDP-43-YFP (A) or total cell lysates (B) were immunoblotted for ubiquitin and TDP-43. (C) A significant increase in MG132-induced ubiquitination of TDP-43-ΔTBD-YFP was detected at an earlier time point compared with TDP-43-WT-YFP, indicating that TDP-43-ΔTBD is more rapidly ubiquitinated than TDP-43-WT. Mean±s.d. of three independent experiments. Two-way ANOVA (*P*<0.0273) and an uncorrected Fisher's exact test for significance. (D,E) Cells expressing TDP-43-WT-YFP were exposed to vehicle (DMSO), MG132 alone or MG132 plus the Tnks-1/2 inhibitor G007-LK (1 or 10 µM). Immunoblots of immunoprecipitated TDP-43-WT (D) and total cell lysate (E) probed for ubiquitin and TDP-43. (F) Co-treatment with 10 µM G007-LK and MG132 significantly increased the levels of ubiquitinated TDP-43-WT-YFP compared with MG132 alone. Mean±s.d. of three independent experiments. One-way ANOVA (*P*=0.0013) followed by a Tukey's test for significance. NS, not significant; *P<0.05, **P<0.01, ***P<0.001.

Given that Tnks-1/2 stabilized TDP-43 (Fig. 3), we examined the ubiquitination levels of TDP-43 upon proteasome inhibition. To do this, we treated cells with control (DMSO) or the proteasome inhibitor MG132 for 2 h or 4 h, then immunoprecipitated either TDP-43-WT-YFP or TDP-43-ΔTBD-YFP and probed for ubiquitin conjugation. Results showed a significant increase in ubiquitinated TDP-43-WT after 4 h of MG132 treatment (Fig. 4A-C). By contrast, a significant increase in MG132-induced ubiquitination of TDP-43-ΔTBD was detected after only 2 h of treatment (Fig. 4A-C), indicating that TDP-43-ΔTBD is more rapidly ubiquitinated than TDP-43-WT. To test whether pharmacological inhibition of Tnks-1/2 regulates MG132-induced ubiquitination of TDP-43-WT-YFP, we treated cells with G007-LK, a highly selective inhibitor of Tnks-1/2 activity with no effect on PARP-1 (Voronkov et al., 2013). Consistent with TDP-43-ΔTBD being more rapidly ubiquitinated than TDP-43-WT (Fig. 4A-C), treatment with the Tnks-1/2 inhibitor G007-LK (10 µM) significantly increased the levels of MG132-induced ubiquitination of TDP-43-WT compared with controls (Fig. 4D-F). A second Tnks-1/2 inhibitor, XAV939, behaved similarly (Fig. S5A-C). The effect of Tnks-1/2 inhibition on MG132-induced ubiquitination of TDP-43 was not additive, as treatment with the Tnks-1/2 inhibitor alone did not lead to ubiquitination of TDP-43-WT-YFP (Fig. S6A,B). Collectively, these data demonstrate that loss of the Tnks-1/2 interaction or reduced Tnks-1/2 activity promotes MG132-induced ubiquitination of TDP-43 and increases proteasomal degradation of the protein. Thus, our data suggest that Tnks-1/2 maintains TDP-43 in a non-ubiquitinated and stabilized state.

### Proteasomal turnover of TDP-43 occurs in the nucleus

**Fig. 5.**
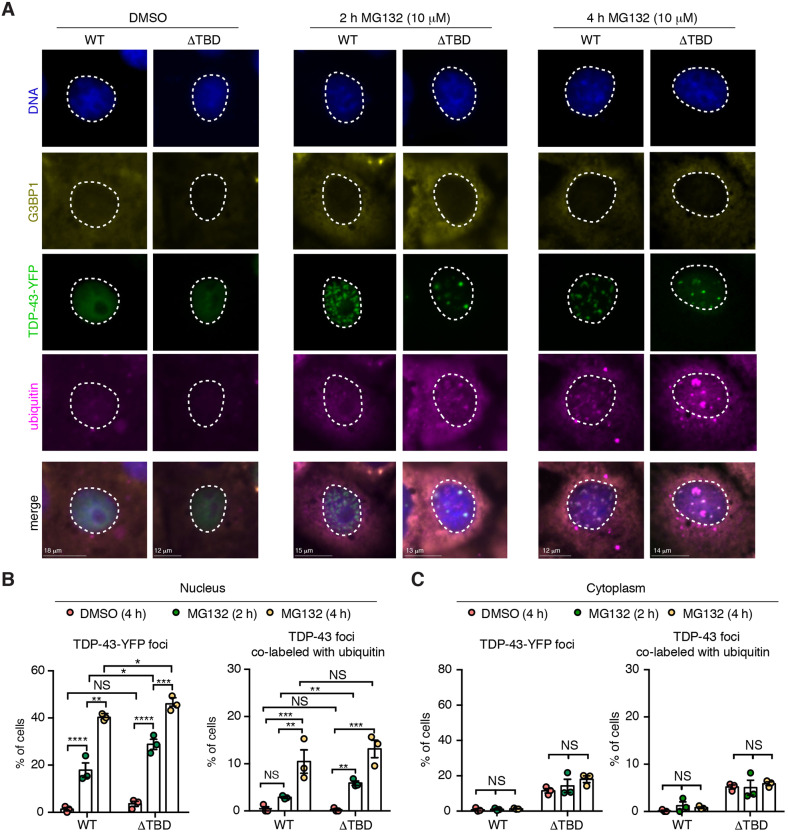
**Deletion of the TBD promotes MG132-induced nuclear ubiquitination of TDP-43.** (A) COS-7 cells expressing TDP-43-WT-YFP and TDP-43-ΔTBD-YFP were treated with vehicle or MG132 for the indicated time periods. Cells were fixed and immunolabeled for the cytoplasmic protein G3BP1 (yellow) and ubiquitin (magenta), and counterstained with Hoechst 33342 (blue). Both TDP-43-YFP proteins formed nuclear foci upon proteasome inhibition (green). Nuclear foci of TDP-43-ΔTBD were co-labeled with ubiquitin by 2 h of MG132 treatment, whereas TDP-43-WT was only co-labeled with ubiquitin by 4 h of MG132 treatment. Nuclei are outlined in white dashed lines. (B) Cells were quantified for the presence of nuclear TDP-43-YFP foci and for nuclear TDP-43-YFP foci that co-labeled with ubiquitin. Both TDP-43 variant proteins (WT and ΔTBD) formed nuclear foci in response to MG132 treatment. Cells were quantified for the presence of nuclear TDP-43-YFP foci that co-labeled with ubiquitin. A significant increase in nuclear TDP-43-ΔTBD foci co-labeled with ubiquitin was detected at an earlier time point compared with TDP-43-WT (2 h versus 4 h). Mean±s.e.m. of three independent experiments. Two-way ANOVA (left graph, *P*<0.0001; right graph, *P*<0.0001) and unpaired *t*-tests. (C) Cells were quantified for the presence of cytoplasmic TDP-43-YFP foci and for cytoplasmic TDP-43-YFP foci that co-labeled with ubiquitin. No increase in cytoplasmic foci formation was observed either for TDP-43-WT or TDP-43-ΔTBD or for cytoplasmic TDP-43-WT or TDP-43-ΔTBD foci co-labeled with ubiquitin in response to MG132 treatment. Mean±s.e.m. of three experiments. Two-way ANOVA (not significant; right graph *P*<0.1931 and left graph *P*=0.672). NS, not significant; *P<0.05, **P<0.01, ***P<0.001, ****P<0.0001.

To gain an understanding of how Tnks-1/2 could lead to stabilization of TDP-43, we examined TDP-43 localization by immunofluorescence. Under normal conditions, both TDP-43-WT-YFP and TDP-43-ΔTBD-YFP localized diffusely to the nucleus. In response to MG132 treatment, both proteins formed nuclear foci that co-labeled with ubiquitin (Fig. 5A,B). However, ubiquitin co-labeling occurred significantly earlier for nuclear TDP-43-ΔTBD foci than for TDP-43-WT foci (2 h versus 4 h of treatment) (Fig. 5A,B). These data are consistent with our finding that TDP-43-ΔTBD is more rapidly ubiquitinated than TDP-43-WT (see Fig. 4) and indicates that proteasome inhibition causes both TDP-43 proteins (WT and ΔTBD) to accumulate in ubiquitin-positive foci in the nucleus.

**Fig. 6.**
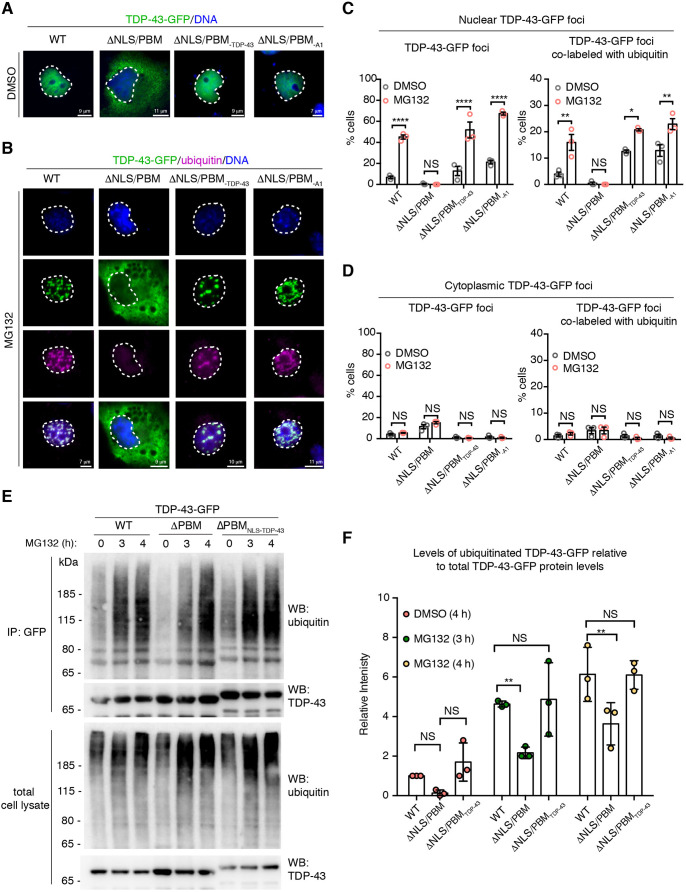
**MG132-treatment led to the formation of nuclear TDP-43 foci that co-label with ubiquitin.** (A) TDP-43-WT-GFP diffusely localizes to the nucleus whereas TDP-43-ΔNLS/PBM-GFP is diffusely cytoplasmic. Addition of an exogenous NLS from either TDP-43 (-ΔNLS/PBM_TDP-43_) or hnRNPA1 (-ΔNLS/PBM_A1_) localizes TDP-43-ΔNLS/PBM-GFP to the nucleus. COS-7 cells were treated with DMSO for 3 h, fixed and counterstained with Hoechst 33342. (B) Treatment with 10 µM MG132 (3 h) led to the formation of nuclear TDP-43-WT-GFP foci that co-labeled with ubiquitin, whereas TDP-43-ΔNLS/PBM-GFP in the cytoplasm remained diffuse. TDP-43-ΔNLS/PBM re-localized to the nucleus by an exogenous NLS, from either TDP-43 (-ΔNLS/PBM_TDP-43_) or hnRNPA1 (-ΔNLS/PBM_A1_), formed MG132-induced nuclear foci that co-labeled with ubiquitin. These data suggest that mutation in the NLS/PBM does not inhibit foci formation. COS-7 cells were immunolabeled for ubiquitin, fixed and counterstained with Hoechst 33342. (C) TDP-43-GFP localized to the nucleus (-WT, -ΔNLS/PBM_TDP-43_ and -ΔNLS/PBM_A1_) forms foci that co-label with ubiquitin upon treatment with 10 µM MG132 (3 h). Cells were quantified for the presence of nuclear TDP-43-GFP foci and the presence of nuclear TDP-43-GFP foci that co-labeled with ubiquitin. Mean±s.e.m. of three independent experiments. Two-way-ANOVA (both graphs, *P*<0.0001) and Tukey's test. (D) Treatment with 10 µM MG132 (3 h) did not increase the percentage of cells with cytoplasmic TDP-43-GFP foci that co-labeled with ubiquitin. Mean±s.e.m. of three independent experiments. Two-way-ANOVA (not significant; right graph, *P*=0.7789, left graph, *P*=0.6644). (E) Cells expressing TDP-43-GFP (-WT, -ΔNLS/PBM or -ΔNLS/PBM_TDP-43_) were exposed to vehicle (DMSO) or MG132 (10 µM). Immunoblots of (top panel) immunoprecipitated TDP-43-GFP or (bottom panel) total cell lysate for ubiquitin and TDP-43. (F) Upon MG132 treatment, the level of ubiquitinated TDP-43-GFP relative to total protein levels was significantly lower for TDP-43-ΔNLS/PBM than for TDP-43-WT and TDP-43-ΔNLS/PBM_TDP-43_. Mean±s.d. of three independent experiments. Two-way ANOVA (*P*<0.0001) and a Dunnett's test. NS, not significant; *P<0.05, **P<0.01, ****P<0.0001.

Curiously, we observed that MG132 treatment did not lead to an increase in the percentage of cells with cytoplasmic foci of TDP-43-WT or TDP-43-ΔTBD, or with cytoplasmic foci of the protein co-labeled with ubiquitin (Fig. 5A,C). This appears to be in contrast to some studies, but consistent with others, and we suggest that this difference is due to differing time periods of MG132 treatment (see Discussion). This result, however, raised the possibility that proteasome inhibition may preferentially promote accumulation of TDP-43 selectively in the nucleus. To explore proteasomal-turnover of TDP-43 in the context of the cellular milieu, we examined the effect of MG132 on a form of TDP-43 that cannot be imported into the nucleus and instead localizes to the cytoplasm. TDP-43 nuclear localization is dependent upon a bipartite nuclear localization sequence (NLS) that also acts as a PAR-binding motif (PBM) in the N-terminal portion of the protein (Fig. S7A); mutation of this region (TDP-43-ΔNLS/PBM) prevents nuclear import and binding to PAR (McGurk et al., 2018a; Winton et al., 2008). Under conditions in which MG132 treatment increased the percentage of cells with nuclear TDP-43-WT-GFP foci [from 9.4±1.3% to 45±3.6% (s.e.m.); Fig. 5C], TDP-43-ΔNLS/PBM-GFP remained diffusely cytoplasmic (Fig. 6B,D; Fig. S7B-D). It is important to note that under the same conditions, the cytoplasmic protein G3BP1 formed cytoplasmic foci in response to MG132 treatment (Fig. S8A,B), which is consistent with previous reports (Mazroui et al., 2007). Thus, our data suggest that, under the conditions tested, TDP-43 localized to the cytoplasm (TDP-43-ΔNLS/PBM) does not respond to MG132.

To determine whether TDP-43-ΔNLS/PBM remained diffuse upon MG132 treatment because of its cytoplasmic localization or, alternatively, because the NLS/PBM mutation impaired the ability of the protein to respond to proteasome inhibition, we generated TDP-43-ΔNLS/PBM-GFP with an exogenous NLS sequence. We compared the bipartite NLS/PBM from the TDP-43 protein (TDP-43-ΔNLS/PBM_TDP-43_) to the proline-tyrosine NLS (PY-NLS) from heterogeneous nuclear ribonucleoprotein A1 (hnRNPA1; TDP-43-ΔNLS/PBM_A1_) (Fig. S7A). The NLS from TDP-43 differs from the PY-NLS not only in amino acid sequence but also in the transport system used to direct proteins to the nucleus. Importin α/β (also known as karyopherin α/β1) directs TDP-43 to the nucleus whereas transportin (also known as karyopherin β2) directs hnRNPA1 to the nucleus (Lee et al., 2006; Winton et al., 2008). Under normal conditions TDP-43-ΔNLS/PBM_TDP-43_ and TDP-43-ΔNLS/PBM_A1_ localized to the nucleus (Fig. 6A) and, upon treatment with MG132, both TDP-43-ΔNLS/PBM_TDP-43_ and TDP-43-ΔNLS/PBM_A1_ formed ubiquitin-labeled nuclear foci (Fig. 6B-D). These data suggest that TDP-43 must be in the nucleus to form MG132-induced nuclear foci. These data also suggest that the NLS/PBM sequence of TDP-43 is not required for the response to MG132 treatment, because the addition of a different NLS sequence (TDP-43-ΔNLS/PBM_A1_) also rescued MG132-induced accumulation of the protein in the nucleus.

To gain a molecular understanding of TDP-43 turnover in the context of the cell, we assessed the ubiquitination levels of immunoprecipitated TDP-43-GFP localized to the nucleus (-WT, -NLS/PBM_TDP-43_) or cytoplasm (-ΔNLS/PBM). Upon MG132 treatment, both nuclear forms of TDP-43 (-WT and -ΔNLS/PBM_TDP-43_) were ubiquitinated (Fig. 6E,F). Although MG132 treatment led to an increase in ubiquitination of cytoplasmic TDP-43 (-ΔNLS/PBM) compared with baseline, the levels were significantly lower than for TDP-43-WT (Fig. 6E,F). This finding indicates that upon MG132 treatment, ubiquitinated TDP-43-GFP localized to the nucleus accumulates more rapidly than ubiquitinated TDP-43-GFP in the cytoplasm. These data further suggest that, in this assay, TDP-43 is preferentially degraded by the nuclear proteasome.

### Tankyrase-1/2 promotes cytoplasmic accumulation of TDP-43

**Fig. 7.**
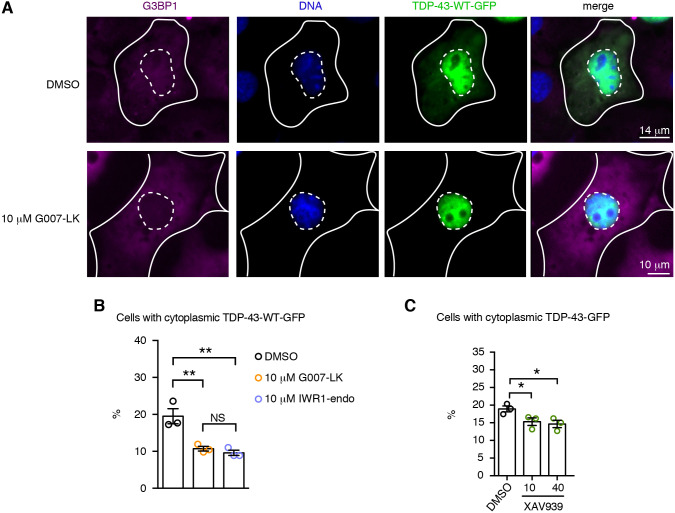
**Tnks-1/2 inhibition promotes nuclear localization of TDP-43.** (A) Treatment with Tnks-1/2 inhibitor G007-LK (10 µM) reduced cytoplasmic accumulation of TDP-43-WT-GFP. Cells were immunostained for the cytoplasmic protein G3BP1 (magenta) and counterstained with Hoechst 33342 (blue). Inner dashed line marks the nuclear boundary and outer solid line marks the cytoplasmic boundary. (B) Treatment of cells with Tnks-1/2 inhibitors G007-LK and IWR1-endo led to a significant reduction in the percentage of cells with cytoplasmic TDP-43-GFP. The percentage of cells with cytoplasmic TDP-43-GFP showing a diffuse GFP signal or GFP-positive puncta was quantified. Mean±s.e.m. of three independent experiments. One-way ANOVA (*P*=0.0032) and a Tukey's tests. (C) Treatment with the Tnks-1/2 inhibitor XAV939 led to a significant reduction in the percentage of cells with cytoplasmic TDP-43-GFP. Mean±s.e.m. of three independent experiments. One-way ANOVA (*P*<0.0004) and a Holm's-Sidak test. NS, not significant; *P<0.05, **P<0.01.

**Fig. 8.**
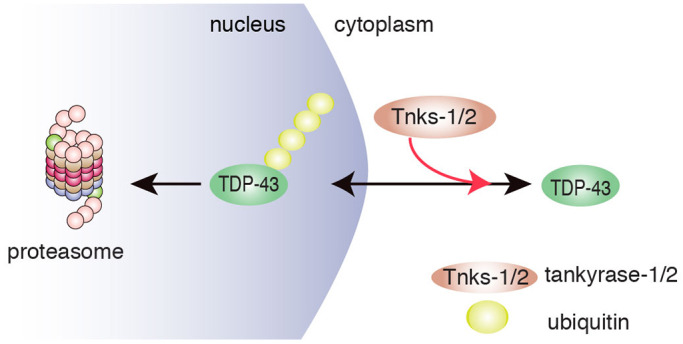
**Model for effects of Tnks-1/2 to modulate the subcellular localization of TDP-43.** The data show that Tnks-1/2 promotes cytoplasmic accumulation of TDP-43, thereby inhibiting access of TDP-43 to the nuclear proteasome. In this way, Tnks-1/2 stabilizes TDP-43 in the cytoplasm. In human disease, accumulation of TDP-43 in the cytoplasm is observed in affected brain cells of >95% of ALS cases and ∼45% of FTD cases. Thus, therapeutic inhibition of Tnks-1/2 in ALS/FTD may maintain TDP-43 in the nucleus where misfolded or mutated forms of the protein can be degraded by the nuclear proteasome.

Previously, we found that Tnks-1/2 regulates the cytoplasmic accumulation of TDP-43 in aging neurons in *Drosophila* and in the cytoplasm of mammalian cells exposed to the chemical stressor arsenite (McGurk et al., 2018a). Furthermore, Tnks-1/2 has been reported to localize to the cytoplasmic face of the nuclear pore complex (Smith and de Lange, 1999). We thus considered that Tnks-1/2 might inhibit turnover of TDP-43 by promoting cytoplasmic accumulation of the protein. To explore this possibility, we examined the localization of TDP-43-WT-GFP in cells treated with highly selective Tnks-1/2 inhibitors (G007-LK and IWR1-endo) that have no reported effect on PARP-1 (Huang et al., 2009; Voronkov et al., 2013). Under control conditions, TDP-43-WT was observed in the cytoplasm of 19.5±2% (s.e.m.) of cells (Fig. 7A,B), and treatment with the Tnks-1/2 inhibitors G007-LK or IWR1-endo significantly (*P*=0.0032) reduced the percentage of cells with observable cytoplasmic TDP-43-WT [from 19.5±2% to 10.7±0.6% and 9.6±0.7% (s.e.m.), respectively] (Fig. 7B). A third inhibitor of Tnks-1/2, XAV939, also reduced the percentage of cells with TDP-43-WT in the cytoplasm (Fig. 7C). Combined, these data indicate that under non-stressed conditions, Tnks-1/2 activity promotes cytoplasmic accumulation of TDP-43-WT. Furthermore, we suggest that, by promoting cytoplasmic accumulation of TDP-43, Tnks-1/2 indirectly inhibits TDP-43 degradation by the nuclear proteasome (Fig. 8), leading to its stabilization.

## DISCUSSION

We have discovered that, in mammalian cells, TDP-43 and Tnks-1/2 proteins interact through a newly defined tankyrase-binding domain in TDP-43. Unlike previously established Tnks-1/2 substrates such as axin, this interaction does not promote ubiquitination and degradation of TDP-43. Instead, the interaction with Tnks-1/2 stabilizes TDP-43, potentially by promoting the cytoplasmic localization of TDP-43. Our data also suggest that ubiquitinated TDP-43 preferentially accumulates as foci in the nucleus. In the context of ALS/FTD, Tnks-1/2 inhibitors, and potentially inhibitors specific for the TDP-43 and Tnks-1/2 interaction, could help maintain TDP-43 in the nucleus, where damaged and misfolded forms of the protein can be turned over by the proteasome. Consistent with this, we demonstrate that pharmacological inhibition of Tnks-1/2 activity protects against TDP-43-associated neurotoxicity in primary rodent neurons.

The tankyrase-binding motif is a loosely conserved motif of RxxΦDG (Guettler et al., 2011; Sbodio and Chi, 2002). The first and last amino acids (R1 and G6) in peptides or fragments that span the tankyrase-binding motif of 3BP2, axin1, RNF146 and IRAP abolish the interaction with Tnks-1/2 and are considered to be invariant amino acids (DaRosa et al., 2018; Guettler et al., 2011; Morrone et al., 2012; Sbodio and Chi, 2002). Mutation analysis of a peptide spanning the TBD of 3BP2 demonstrated that at position 4, glycine, proline, alanine and cysteine are preferred whereas isoleucine, leucine and valine prevent Tnks-1/2 binding to the 3BP2 peptide (Guettler et al., 2011). This appears in contrast with our detailed mutational analysis of the tankyrase binding domain in TDP-43, which shows that mutation of R1 or G6 has little to no effect on the interaction between TDP-43 and Tnks-1/2. Furthermore, not only is position 4 of the TBD an isoleucine, but mutation of this amino acid in the full-length form of TDP-43 abolishes the interaction with Tnks-1/2. Thus, we suggest that TDP-43 harbors a tankyrase-binding motif with non-canonical features. It is important to note that non-canonical and extended tankyrase-binding motifs have been identified and include an extra two amino acids after position 6 in 3BP2 and motifs with additional amino acids between positions 1 and 2 in APC2, axin1 and RNF146 (Croy et al., 2016; DaRosa et al., 2018; Guettler et al., 2011; Morrone et al., 2012). Furthermore, the second tankyrase-binding motif in axin is not dependent on R1 (Morrone et al., 2012) and tankyrase-binding motifs in several other proteins harbor amino acids that are not tolerated at the fourth position in the 3BP2 peptide including a valine (motif 1 of axin 1, motif 2 of RNF146, motif 1 of APC2 and motif 3 of PEX14) and a leucine (motif 2 of MDC1) (Croy et al., 2016; DaRosa et al., 2018; Li et al., 2017b; Morrone et al., 2012; Nagy et al., 2016). Thus, in the context of different proteins or perhaps context-dependent functions of Tnks-1/2, the constraints on the consensus of the tankyrase-binding motif might differ.

Functionally, Tnks-1/2 has a central role in PARylation-dependent ubiquitination, a process that can lead to proteasomal degradation of target proteins such as axin, 3BP2, HIPPO, PARP-1, PTEN, TRF1 and YAP1 (Bhardwaj et al., 2017; Callow et al., 2011; Chang et al., 2003; Croy et al., 2016; Feng et al., 2014; Huang et al., 2009; Levaot et al., 2011; Li et al., 2015; Zhang et al., 2011). Surprisingly, our studies suggest that Tnks-1/2 stabilizes TDP-43. These results encompass pharmacological inhibition of Tnks-1/2 activity on endogenous TDP-43 protein and expression of exogenous TDP-43-WT-YFP forms with mutational variants unable to associate with Tnks-1/2 (-ΔTBD, -H166 and -I168). Although our findings may appear in contrast to the examples of Tnks-1/2 promotion of PAR-dependent proteolysis, it is important to note that upon Wnt stimulation, Tnks-1/2 function stabilizes axin at the cell membrane, promoting the association of axin with the Wnt receptor (Wang et al., 2016; Yang et al., 2016). Finally, our previous finding that the Tnks-1/2 homologue is a dose-sensitive modifier of TDP-43 neurotoxicity in *Drosophila* (McGurk et al., 2018a) may in part be explained by the ability of Tnks-1/2 activity to stabilize TDP-43 protein levels.

The mechanism by which Tnks-1/2 stabilizes TDP-43 may include cellular localization of the protein. Using a combination of biochemistry and immunofluorescence, we observed that upon proteasome inhibition, TDP-43 primarily accumulates in the nucleus and is more rapidly ubiquitinated than cytoplasmic TDP-43. Our data, presented here and previously (McGurk et al., 2018a), suggest that Tnks-1/2 promotes cytoplasmic accumulation of TDP-43. We hypothesize that, in doing so, Tnks-1/2 may either sequester TDP-43 from the nuclear proteasome or inhibit nuclear import of the protein, which ultimately leads to stabilization (Fig. 8). Tnks-1/2 activity is known to regulate protein localization, for example by promoting axin localization to the Wnt receptor, or alternatively by directing its degradation (De Rycker and Price, 2004; Mariotti et al., 2016; Wang et al., 2016; Yang et al., 2016). Whether the regulation of TDP-43 localization by Tnks-1/2 is direct or indirect and whether this involves modification of TDP-43 (e.g. by PARylation) remain to be established.

Impairment of the proteasome has been hypothesized to be a disease-causing mechanism in ALS/FTD (Scotter et al., 2015; Taylor et al., 2016); thus, many studies have examined TDP-43 cellular localization upon proteasome inhibition with MG132. Treatment with MG132 for extended time periods (ranging from 12 to 72 h) leads to diffuse accumulation of TDP-43 in the cytoplasm (Klim et al., 2019; van Eersel et al., 2011; Walker et al., 2013) or cytoplasmic aggregates of TDP-43 (Huang et al., 2014; Li et al., 2017a; Scotter et al., 2014). Our data demonstrate that treatment with MG132 for short periods of time (3-5 h) leads to nuclear foci formation of TDP-43-WT, whereas TDP-43 localized to the cytoplasm (TDP43-ΔNLS/PBM) remains diffuse. Our findings are consistent with previous studies showing that TDP-43-WT forms nuclear foci in cells treated with MG132 for 8 h (Wang et al., 2010) and that TDP-43 with mutation the NLS/PBM remains diffuse upon treatment with MG132 for 6 h (Nonaka et al., 2009). Thus, we propose that, upon proteasomal inhibition, TDP-43 accumulation in the nucleus occurs earlier than in the cytoplasm. Our data further suggest that turnover of TDP-43 by the nuclear proteasome is important for regulating TDP-43 degradation.

An impact on global protein levels is thought to be involved in ALS/FTD. Many disease-associated mutations occur in proteins that function in protein turnover such as the UPS and autophagy, including C9orf72, charged multivesicular body protein 2b, optineurin, sequestrome 1, serine/threonine protein kinase TBK1, ubiquilin 2 and valosin-containing protein (VCP), suggesting broad impairment of protein turnover in neurodegenerative disease (Balendra and Isaacs, 2018; Gao et al., 2017; Taylor et al., 2016). Furthermore, altered TDP-43 protein levels have also been implicated in disease. For example, mRNA levels for TDP-43 are upregulated in post-mortem tissue from patients with ALS/FTD as well as in a knock-in mouse model for the Q331K disease-causing mutation in TDP-43 (Gitcho et al., 2009; Mishra et al., 2007; White et al., 2018). In post-mortem tissue, TDP-43 protein abnormally accumulates in ubiquitin inclusions in the cytoplasm of affected neurons and glia, suggesting that the affected cells cannot remove and degrade TDP-43 in the cytoplasm (Arai et al., 2006; Mackenzie et al., 2007; Neumann et al., 2007). In patient fibroblasts (sporadic and those harboring a G_4_C_2_-hexanucleotide expansion in *C9orf72* or mutation in TDP-43), the levels of cytoplasmic TDP-43 and of total TDP-43 protein are significantly higher than in control cells (Lee et al., 2019; Sabatelli et al., 2015). Additionally, upon proteasome inhibition, TDP-43 protein levels remain unaltered in these ALS/FTD patient fibroblasts (Lee et al., 2019), suggesting that UPS turnover of TDP-43 is impaired. It is intriguing to postulate that UPS-mediated turnover of TDP-43 is impaired in ALS/FTD patient fibroblasts because TDP-43 accumulates in the cytoplasm and thus is sequestered from the nuclear proteasome.

There are no effective treatments for ALS/FTD and related disorders such as Parkinson's disease. In ALS, nuclear PAR is elevated in motor neurons of post-mortem spinal cord tissue and total PAR is elevated in the cerebrospinal fluid of patients with Parkinson's disease (Kam et al., 2018; McGurk et al., 2018c). These findings suggest that inhibiting PARP activity may have therapeutic potential. Similar to inhibition of Tnks-1/2, chemical inhibition of nuclear PARP-1/2 activity reduces accumulation of TDP-43 in stress foci in the cytoplasm (McGurk et al., 2018a). Thus, nuclear PARP activity may promote nuclear export of TDP-43, which may also lead to reduced turnover of the protein. Finding agents that regulate the levels of TDP-43 is important for modulating TDP-43 protein homeostasis, as well as removing misfolded and possibly toxic forms of the protein that accumulate in affected brain regions. We propose that inhibition of Tnks-1/2, which regulates TDP-43 stability and neurotoxic properties, is a potential therapeutic target for ALS/FTD and related disorders.

## MATERIALS AND METHODS

### Rat cortical neuron culture and neurotoxicity assay

Rat cortical neurons were from embryos isolated from female Sprague Dawley wild rats that were 16-18 days pregnant (Neuron R Us, neuron service center, University of Pennsylvania). About 100,000 neurons were plated out on poly-D-lysine coated coverslips (12 mm diameter and thickness #1; Neuvitro, Vancouver, WA, Canada) in neurobasal medium supplemented with serum-free B27, penicillin streptomycin and Glutamax (all from ThermoFisher Scientific Waltham, MA, USA). Neurons were cultured at 37°C with 5% CO_2_. Three times per week, half of the medium was removed and replaced with fresh prewarmed medium. The primary neurons were infected at 14-17 days *in vitro* with HSV-TDP-43 or HSV-LacZ with either the Tnks-1/2 inhibitor G007-LK (SelleckChem, Houston, TX, USA) or DMSO (Sigma Aldrich, St Louis, MI, USA). Every 2 days, half of the medium was removed and replaced with fresh medium containing G007-LK or DMSO. The drug-containing medium was made up at 2× concentration so that when it was diluted twofold in the well it was of the appropriate concentration. At 7 days post infection, the neurons were fixed in 4% paraformaldehyde, blocked in 10% normal donkey serum (Sigma Aldrich) in Tris-buffered saline containing 0.05% Tween 20 (TBST) (ThermoFisher Scientific) for 1 h at room temperature, and immunostained overnight at 4°C with antibodies directed to the neuronal marker tubulin β-3 chain (1:500; Abcam, Cambridge, UK). After three sets of 5 min washes in TBST, neurons were incubated with mouse AlexaFluor 488 (1:500; ThermoFisher Scientific) in the dark for 1 h at room temperature. Neurons were washed three times (5 min each) with TBST, counterstained for 15 min with 1 µg/ml Hoechst 33342 (ThermoFisher Scientific), washed in deionized H_2_O and mounted in ProLong Diamond (ThermoFisher Scientific). Five images (10× magnification) were captured from each coverslip and the remaining neuronal cell bodies in each image counted. Each condition was repeated three times on three independent cultures, each from a different pregnant rat.

### Mammalian cells and culture details

COS-7 cells originally purchased by ATCC were a gift from Virginia M. Lee (University of Pennsylvania). Prior to purchase, the COS-7 cells were authenticated by ATCC. HEK293T cells were kindly provided by Aaron Gitler (Stanford University). COS-7 cells were routinely grown in Dulbecco's modified Eagle's medium (DMEM) containing high glucose and L-glutamine (ThermoFisher Scientific), 10% filter-sterile FBS (Sigma Aldrich) and penicillin-streptomycin (ThermoFisher Scientific). HEK-293T cells were grown in DMEM with high glucose, L-glutamine and sodium pyruvate (ThermoFisher Scientific), 10% filter-sterile FBS (Sigma Aldrich) and penicillin-streptomycin (ThermoFisher Scientific). Cells were grown at 37°C with 5% CO_2_; a water bath was used for humidification. Cells were washed with Dulbecco's PBS without calcium or magnesium (ThermoFisher Scientific) and trypsinized in trypsin with 0.25% EDTA (ThermoFisher Scientific). No commonly misidentified cell lines were used.

### Identification of the tankyrase-binding domain

We computationally aligned the tankyrase-binding motif (RxxΦDG) to the human TDP-43 protein sequence using the PATTINPROT search engine (Combet et al., 2000). To map the TBD to the reported NMR structures of RRM1 and RRM2 of TDP-43 (4BS2) (Lukavsky et al., 2013), we used the open source Java viewer ‘FirstGlance in Jmol’.

### Plasmids

Human TDP-43-WT-YFP in pcDNA3.2 was described previously (Elden et al., 2010). TDP-43-WT-GFP and TDP-43-ΔPBM both in pcDNA3-C-eGFP were described previously (McGurk et al., 2018a).

TDP-43-ΔTBD, -D169G, -R165A, -H166A, -M167A, -I168A, -D169A and -G170A were made by performing site-directed mutagenesis (QuikChange II XL) on TDP-43-WT-YFP using the following forward primers (FP) and reverse primers (RP): ΔTBD, FP 5′-CAAGTGAAAGTAATGTCACAGCGATGGTGTGACTGCAAACTTCC-3′ and RP 5′-GGAAGTTTGCAGTCACACCATCGCTGTGACATTACTTTCACTTG-3′; D169G, FP: 5′-TCACAGCGACATATGATAGGTGGACGATGGTGTGACTGC-3′ and RP: 5′-GCAGTCACACCATCGTCCACCTATCATATGTCGCTGTGA-3′; R165A, FP 5′-CATCGTCCATCTATCATATGTGCCTGTGACATTACTTTCACTTG-3′ and RP 5′-CAAGTGAAAGTAATGTCACAGGCACATATGATAGATGGACGATG-3′; H166A, FP 5′-CACCATCGTCCATCTATCATAGCTCGCTGTGACATTACTTTCAC-3′ and RP 5′-GTGAAAGTAATGTCACAGCGAGCTATGATAGATGGACGATGGTG-3′; M167A, FP 5′-CACACCATCGTCCATCTATCGCATGTCGCTGTGACATTACTT-3′ and RP 5′-AAGTAATGTCACAGCGACATGCGATAGATGGACGATGGTGTG-3′; I168A, FP 5′-GTCACACCATCGTCCATCTGCCATATGTCGCTGTGACATT-3′ and RP 5′-AATGTCACAGCGACATATGGCAGATGGACGATGGTGTGAC-3′; D169A, FP 5′-GTCACACCATCGTCCAGCTATCATATGTCGCTGT-3′ and RP 5′-ACAGCGACATATGATAGCTGGACGATGGTGTGAC-3′; G170A, FP 5′-TGCAGTCACACCATCGTGCATCTATCATATGTCGC-3′ and RP 5′-GCGACATATGATAGATGCACGATGGTGTGACTGCA-3′.

The DNA sequence corresponding to the NLS of TDP-43 (TDP-43-ΔPBM-NLS_TDP-43_) or the NLS of hnRNPA1 (TDP-43-ΔPBM-NLS_A1_) was cloned immediately upstream of the stop site in TDP-43-ΔPBM-GFP in pcDNA3-C-eGFP by GenScript, Piscataway, NJ, USA. The following DNA sequences were subcloned: TDP-43-ΔPBM-NLS_TDP-43_, 5′-AAAAGAAAAATGGATGAGACAGATGCTTCATCAGCAGTGAAAGTGAAAAGA-3′; TDP-43-ΔPBM-NLS_A1_, 5′-AATCAGTCTTCAAATTTTGGACCCATGAAGGGAGGAAATTTTGGAGGCAGAAGCTCTGGCCCCTATGGCGGTGGAGGCCAATACTTTGCAAAACCACGAAACCAAGGTGGCTAT-3′.

All plasmid inserts were sequenced and confirmed to be free of any errors.

### Co-immunoprecipitation and immunoblotting

To examine the interaction between endogenous TDP-43 and Tnks-1/2, 2.5 µg of control mouse IgG (Santa Cruz Biotechnology, Dallas, TX, USA) and 2.5 µg mouse anti-TDP-43 (monoclonal antibody ID: 5028) (Kwong et al., 2014) were coupled to 50 µl Protein G dynabeads (ThermoFisher Scientific). COS-7 and HEK293T cells were each grown to confluency in T75 flasks overnight and lysed the following day (see below for lysis method). For co-immunoprecipitation of transfected TDP-43-YFP and endogenous Tnks-1/2, 2.5 µg mouse IgG (Santa Cruz Biotechnology) and 2.5 µg mouse anti-GFP (3E6, ThermoFisher Scientific) were coupled to 50 µl Protein G dynabeads (ThermoFisher Scientific). COS-7 and HEK293T cells were each seeded at a density of 2.7×10^6^ cells in T75 flasks and left overnight. Cells were transfected with 8 µg plasmid DNA, 8 µl PLUS reagent and 28 µl LTX in 1600 µl OPTIMEM I in DMEM with high glucose and L-glutamine (all ThermoFisher Scientific) and 10% FBS (Sigma Aldrich). For all experiments, cells were trypsinized in trypsin with 0.25% EDTA (ThermoFisher Scientific). Cells were pelleted, washed in Dulbecco's PBS without calcium and without magnesium (ThermoFisher Scientific) and resuspended in 1 ml ice-cold Pierce IP Lysis Buffer (ThermoFisher Scientific) containing Halt Protease Inhibitor Single-Use Cocktail (ThermoFisher Scientific). For all co-immunoprecipitations, lysates from each T75 flask (∼8.4×10^6^ cells) were incubated on ice for 10 min, passed three times through a 20G1½ syringe attached to a 1 ml syringe (BD Biosciences, San Jose, CA, USA), transferred to centrifuge tubes, rotated at 15 rpm for 10 min at 4°C and centrifuged at 16,873 ***g*** for 10 min at 4°C. The supernatant was collected, the pellet discarded and 25 µl of lysate removed for input. The remaining lysate was divided in half (500 µl) for incubation with IgG or anti-TDP-43-coupled beads. The reaction volumes were made up to 1 ml with lysis buffer containing protease inhibitor and incubated with the antibody-coupled beads for 18 h at 4°C with 15 rpm rotation. The beads were washed three times by removing the supernatant, adding 500 µl lysis buffer containing protease inhibitor, briefly resuspending the beads, placing the tube on the magnet and immediately removing the buffer. Elution was performed at 95°C for 5 min in 40 µl 1× LDS Sample Buffer (ThermoFisher Scientific) containing 5% β-mercaptoethanol (Sigma Aldrich). Input samples (10 µl) were denatured at 95°C for 5 min in 1× LDS Sample Buffer containing 5% β-mercaptoethanol in a total volume of 20 µl.

Eluates and inputs were electrophoresed (10 µl of the eluate and 10 µl of input were loaded for the detection of TDP-43, and 13 µl eluate and 10 µl input for the detection of Tnks-1/2) on NuPAGE 4-12% Bis-Tris gels (ThermoFisher Scientific) in NuPAGE MES or MOPS buffer (ThermoFisher Scientific). Protein was transferred onto 0.45 µm nitrocellulose membranes (BioRad) using the XCell II Blot Module (ThermoFisher Scientific), in 1× Transfer Buffer (ThermoFisher Scientific) at 20 V for 105 min. Membranes were blocked in TBST (ThermoFisher Scientific) containing 5% non-fat dry milk (LabScientific, Danvers, MA, USA) for 1 h at room temperature, then incubated in primary antibody in TBST overnight at 4°C. Membranes were washed 5×5 min in TBST, incubated in secondary antibody in TBST for 1 h at room temperature, washed 5×5 min in TBST and covered in ECL Prime detection reagent (Amersham, Little Chalfont, UK) for 5 min in the dark. Rabbit anti-TDP-43 (1:10,000; Proteintech, Rosemont, IL, USA, Cat# 10782-2-AP, RRID: AB_615042), rabbit anti-Tnks-1/2 (1:500; Santa Cruz Biotechnology, Dallas, TX, USA, Cat# sc-8337, RRID:AB_661615) and goat anti-rabbit HRP (1:5000; EMD Millipore, Burlington, MA, USA, Cat# 115-035-146, RRID:AB_11212848) were used.

### Immunoprecipitation and immunoblotting

COS-7 cells were seeded at a density of 2.7×10^5^ cells in a six-well plate overnight in DMEM with high glucose and L-glutamine (ThermoFisher Scientific), 10% FBS (Sigma Aldrich) and penicillin-streptomycin (ThermoFisher Scientific). Each well was transfected with 2.5 µg plasmid DNA, 2.5 µl PLUS reagent and 7.9 µl LTX in 400 µl OPTIMEM I (all from ThermoFisher Scientific) in DMEM with high glucose (ThermoFisher Scientific) and 10% FBS (Sigma Aldrich). Control IgG (2.5 µg; Santa Cruz Biotechnology) and anti-GFP-3E6 (2.5 µg; ThermoFisher Scientific) were coupled to 50 µl Protein G dynabeads (ThermoFisher Scientific). For MG132-induced ubiquitination of TDP-43, cells were treated with 10 µM MG132 (Sigma Aldrich) or an equivalent volume of DMSO (Sigma Aldrich) at 23 h post-transfection for the indicated times. Treatment with the DMSO control always matched the longest MG132 treatment. For MG132-induced ubiquitination of TDP-43 in the presence or absence of Tnks-1/2 inhibitor, cells were treated with 10 µM MG132 (Sigma Aldrich) combined with either DMSO (Sigma Aldrich) or XAV939, G007-LK or IWR1-endo (all SelleckChem, Houston, TX, USA) at the indicated times and concentrations. Cells were lysed by adding 300 µl ice-cold RIPA buffer (Cell Signaling, London, UK) containing Halt Protease Inhibitor Single-Use Cocktail (ThermoFisher Scientific) and 5 mM *N*-ethylmaleimide (NEM) (ThermoFisher Scientific) to each well and incubating the plate on a platform shaker at medium speed for 10 min at 4°C. Lysates were passed three times through a 20G1½ 1 ml syringe (BD Biosciences, San Jose, CA, USA), transferred to centrifuge tubes and rotated at 15 rpm for 10 min at 4°C. Lysates were centrifuged for 10 min at 16,873 ***g*** and 4°C. Then, 25 µl of lysate was removed for input and the remaining lysate was made up to 1 ml and incubated with the antibody-coupled beads for 18 h at 4°C with rotation.

Beads were washed three times in 500 µl lysis buffer for 10 min at 4°C with 15 rpm rotation. Elution was performed at 95°C for 5 min in 40 µl 1× LDS Sample Buffer (ThermoFisher Scientific) containing 5% β-mercaptoethanol (Sigma Aldrich). Input samples (10 µl) were denatured at 95°C for 5 min in 1× LDS Sample Buffer (ThermoFisher Scientific) containing 5% β-mercaptoethanol (Sigma Aldrich) in a total volume of 20 µl. Eluates and inputs were electrophoresed (10 µl of the eluate and 10 µl input were loaded for the detection of TDP-43, 13 µl of the eluate and 10 ml of input for the detection of ubiquitin) on NuPAGE 4-12% Bis-Tris gels (ThermoFisher Scientific) in NuPAGE MES or MOPS buffer (ThermoFisher Scientific). Protein was transferred onto 0.45 µm nitrocellulose membranes (BioRad, Hercules, CA, USA) using the XCell II Blot Module (ThermoFisher Scientific), in 1× Transfer Buffer (ThermoFisher Scientific) and run at 30 V for 75 min (note that gels to be transferred to membranes for blotting of ubiquitin were soaked in transfer buffer for 5 min prior to wet transfer). Membranes were blocked in TBST containing 5% non-fat dry milk (LabScientific) for 1 h at room temperature, then incubated in primary antibody in TBST overnight at 4°C. Membranes were washed 5×5 min in TBST, incubated in secondary antibody in TBST for 1 h at room temperature, washed 5×5 min in TBST and covered in ECL Prime detection reagents (Amersham) for 5 min in the dark. Rabbit anti-TDP-43 (1:10,000; Proteintech, Cat# 10782-2-AP, RRID: AB_615042), rabbit anti-ubiquitin (1:1000; Cell Signaling, London, UK Technology, Cat# 3933, RRID: AB_2180538) and goat anti-rabbit HRP (1:5000; EMD Millipore, Cat# 115-035-146, RRID:AB_11212848) were used.

### Cycloheximide pulse chase

To examine the stability of TDP-43-YFP, COS-7 cells were seeded at a density of 2.7×10^5^ cells in a six-well plate overnight in DMEM with high glucose (ThermoFisher Scientific), 10% FBS (Sigma Aldrich) and penicillin-streptomycin (ThermoFisher Scientific). The following day, each well was transfected with 2.5 µg plasmid DNA, 2.5 µl PLUS reagent and 7.9 µl LTX in 400 µl OPTIMEM I in DMEM with high glucose and L-glutamine (all ThermoFisher Scientific) and 10% FBS (Sigma Aldrich). Cells were treated with 100 µg/ml cycloheximide (Sigma Aldrich) in DMEM with high glucose and L-glutamine (all ThermoFisher Scientific) containing 10% FBS (Sigma Aldrich) and penicillin-streptomycin (ThermoFisher Scientific) starting at 24 h after transfection. The media with DMSO (ThermoFisher Scientific) or 100 µg/ml cycloheximide (Sigma Aldrich) were replaced every 24 h during this period. Cells were lysed at 0, 24 and 48 h (see paragraph below for cell lysis). To examine the stability of endogenous TDP-43, a six-well plate was inoculated with COS-7 cells at a seeding density of 5.6×10^5^ cells. Cells were grown in DMEM with high glucose and L-glutamine (ThermoFisher Scientific) containing 10% FBS (Sigma Aldrich) and penicillin-streptomycin (ThermoFisher Scientific) in six-well plates overnight. Cells were treated with 100 µg/ml cycloheximide (Sigma Aldrich) in DMEM containing high glucose and L-glutamine (ThermoFisher Scientific) and 10% FBS (Sigma Aldrich) with penicillin-streptomycin (ThermoFisher Scientific) and lysed (see paragraph below) 1 h after drug treatment.

Cells were lysed by adding 300 µl ice-cold RIPA Buffer (Cell Signaling, London, UK) containing Halt Protease Inhibitor Single-Use Cocktail (ThermoFisher Scientific) and 5 mM *N*-ethylmaleimide (NEM) (ThermoFisher Scientific) and incubated on a platform shaker at medium speed for 10 min at 4°C. Lysates were then passed three times through a 20G1½ 1 ml syringe (BD Biosciences), transferred to centrifuge tubes for 15 rpm rotation for 10 min at 4°C and centrifuged for 10 min at 16,873 ***g*** and 4°C. Inputs were denatured at 95°C for 5 min in 20 µl 1× LDS Sample Buffer (ThermoFisher Scientific) with 5% β-mercaptoethanol (Sigma Aldrich) and run (5 µl) on NuPAGE 4-12% Bis-Tris gels in NuPAGE MES or MOPS buffer (all ThermoFisher Scientific). Wet transfer was performed using 0.45 µm nitrocellulose membranes (BioRad) using the XCell II Blot Module (ThermoFisher Scientific) in 1× Transfer Buffer (ThermoFisher Scientific), run at 20 V for 105 min. Membranes were blocked in TBST (ThermoFisher Scientific) with 5% non-fat dry milk (LabScientific) for 1 h at room temperature, then incubated in primary antibody in TBST overnight at 4°C. Membranes were washed 5×5 min in TBST, incubated in secondary antibody in TBST for 1 h at room temperature, washed 5×5 min in TBST and covered in ECL Prime detection reagent (Amersham) for 5 min in the dark. Rabbit anti-TDP-43 (1:10,000; Proteintech, #10782-2-AP, RRID: AB_615042) and goat anti-rabbit HRP (1:5000; EMD Millipore, #115-035-146, RRID: AB_11212848) were used.

### Immunofluorescence

Immunofluorescence was carried out in a 24-well format. Cells (COS-7 and HEK293T) were seeded at a density of 60,000 cells per well onto glass coverslips (Neuvitro) in DMEM with high glucose and L-glutamine (ThermoFisher Scientific) and 10% FBS (Sigma Aldrich) with penicillin-streptomycin (ThermoFisher Scientific) and incubated overnight at 37°C with 5% CO_2_; a water bath was used for humidification. The following day (∼18 h later), each well was transfected with 500 ng of plasmid DNA, 1.75 µl lipofectamine LTX and 0.5 µl PLUS reagent in 100 µl of OPTIMEM I (all ThermoFisher Scientific). At 21 h post transfection, cells were treated with 10 µM MG132 (Sigma Aldrich) for the indicated amount of time. Treatment with the DMSO control always matched the longest MG132 treatment. For localization studies in the presence of Tnks-1/2 inhibitors, cells were transfected in the presence of the indicated amount of G007-LK or IWR1-endo (both SelleckChem) and fixed for 21 h post-transfection. Cells were fixed in 4% paraformaldehyde (Electron Microscopy Sciences, Hatfield, PA, USA) in 50 mM HEPES pH 7.4, 150 mM NaCl, 1 mM MgCl_2_ and 1 mM EGTA (all Sigma Aldrich). Fixed cells were permeabilized by three treatments of 3 min in 100 mM PIPES, 1 mM MgCl_2_, 10 mM EGTA pH 6.8 and 0.1% Triton-X 100 (PEM-T buffer) (all Sigma Aldrich). Cells were blocked in 10% normal donkey serum (Sigma Aldrich) in TBST (ThermoFisher Scientific). Primary antibodies in TBST (ThermoFisher Scientific) were applied overnight at 4°C in a humidified chamber. Cells were washed three times in PEM-T buffer (3 min each); then, secondary antibody in TBST (ThermoFisher Scientific) was applied for 45 min at room temperature and in the dark. Cells were washed three times in TBST (3 min each), stained with 1 µg/ml Hoechst 33342 (ThermoFisher Scientific) for 15 min, washed in deionized H_2_O and mounted in ProLong Diamond (ThermoFisher Scientific). All experiments were performed at least three independent times.

Primary antibodies used were rabbit anti-G3BP1 (1:1000; ThermoFisher Scientific, #PA5-29455, RRID: AB_2546931) and mouse anti-ubiquitin (1:300; Cell Signaling Technology, London, UK, #3936, RRID: AB_331292). Secondary antibodies (all 1:500; ThermoFisher Scientific) were goat anti-rabbit Alexa Fluor 647 (#A-212245, RRID: AB_2535813); goat anti-rabbit Alexa Fluor 594 (#A11036, RRID:AB_141359); goat anti-mouse Alexa Fluor 647 (#21236, RRID:AB_141725); and donkey anti mouse Alexa Fluor 594 (#21203 RRID: AB_141633).

### Image acquisition and quantification

All imaging was performed on fixed cells at 18-23°C on a Leica DMI6000, widefield epifluorescent microscope (Leica Microsystems, Buffalo Grove, IL, USA). To quantify MG132-induced foci, four or five independent images at 20× were captured. The number of transfected cells was quantified in each image; the number of transfected cells with either nuclear or cytoplasmic foci was counted and the number of cells with nuclear or cytoplasmic TDP-43 foci that co-labeled with ubiquitin was counted and the percentage calculated. To compare the effect of MG132 on TDP-43-WT-YFP versus TDP-43-ΔTBD-YFP, 139-298 transfected cells were quantified per condition. To compare the effect of MG132 on TDP-43-WT-GFP, TDP-43-ΔNLS/PBM-GFP, TDP-43-ΔNLS/PBM_TDP-43_-GFP and TDP-43-ΔNLS/PBM_A1_-GFP, 129-514 transfected cells were quantified per condition. To quantify the effect of Tnks-1/2 inhibition on TDP-43-GFP localization, 4-5 independent images at 20× were captured at the same exposure time. Cells with diffuse cytoplasmic GFP and/or GFP foci in the cytoplasm were scored as cells with cytoplasmic TDP-43-GFP. Up to 540 transfected cells were quantified per condition. All experiments were repeated at least three independent times and the mean±s.e.m. calculated.

### Statistical analysis

Each graph gives the mean (±s.e.m. or s.d.). *n* is the number of biological repeats and is indicated in each figure legend. Student's *t-*tests, one-way ANOVA, two-way ANOVA and multiple comparison tests were performed, as indicated in each figure legend. Significance was set at *P*<0.05; values for asterisks are presented in each legend. All statistical analyses were carried out using Graphpad prism6 software (GraphPad software, San Diego, CA, USA).

## Supplementary Material

Supplementary information

Reviewer comments
