# Supplementary material for: TDP-43, a protein central to amyotrophic lateral sclerosis, is destabilized by tankyrase-1 and -2
Source: J Cell Sci. 2020 Jun 23;133(12):jcs245811. doi: 10.1242/jcs.245811 (PMC7328137; doi:10.1242/jcs.245811)
Supplement: Supplementary information [file joces-133-245811-s1.pdf]

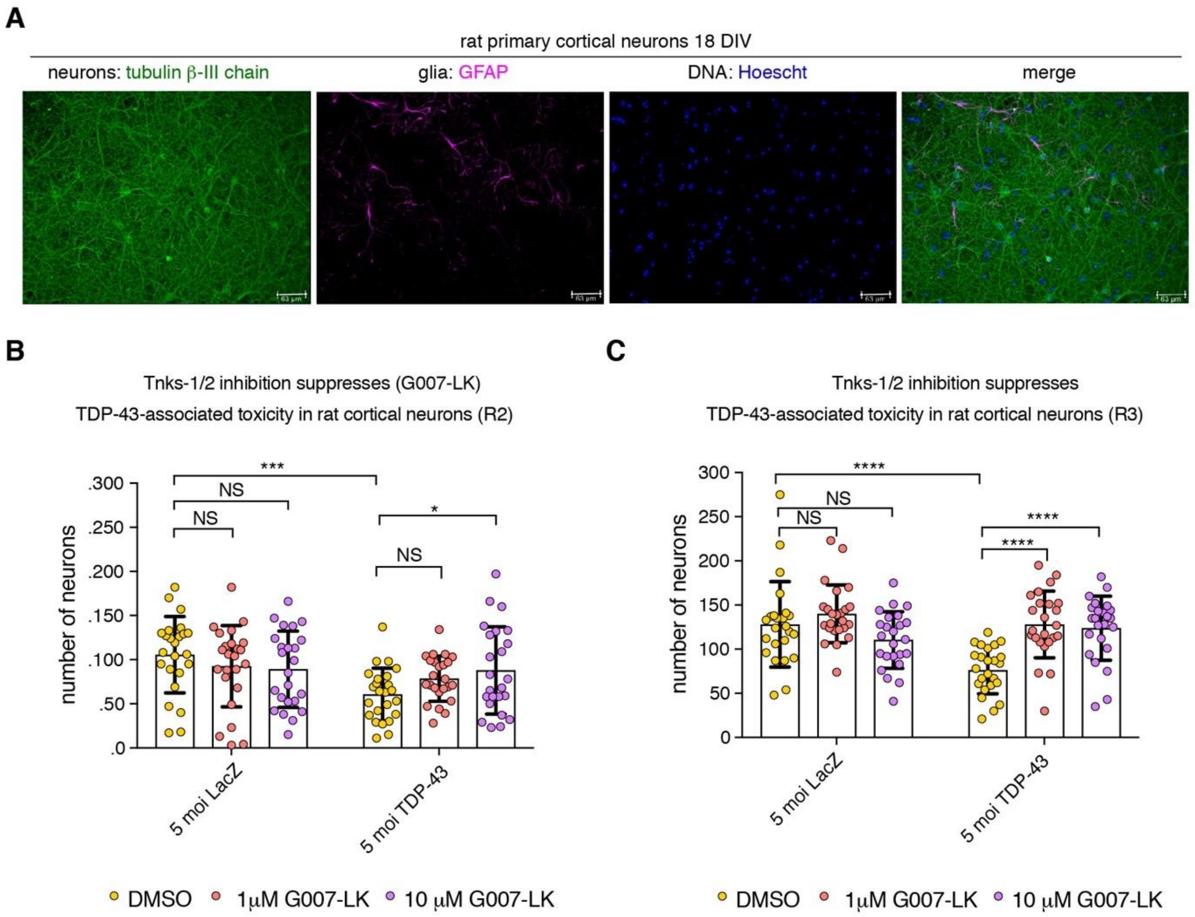

Figure S1: Rat primary cortical neurons and quantification of neuronal cultures infected with TDP-43$_{HSV}$ or LacZ$_{HSV}$ and treated with either vehicle control or the Tnks-1/2 inhibitor G007-LK.

A. Representative images of rat primary cortical neuron cultures at 18 days in vitro (DIV). Cultures were immunolabelled with the neuronal marker tubulin β-III chain (green) and the glial marker GFAP (magenta) and counterstained with Hoechst (blue).

B-C. Viral infection with HSV-TDP-43 at 5 moi resulted in a significant loss in cortical neurons compared to the HSV-LacZ control. Co-treatment with the Tnks-1/2 inhibitor G007-LK (at 1 μM and 10 μM) significantly suppressed TDP-43-associated neuronal loss. Each graph is data from an independent biological repeat. Graphs show individual data points and the mean±s.d. Two-way ANOVA and Dunnet's test were used to reveal pairwise significance (*P< 0.05, *** P< 0.001, ****P< 0.0001, NS: not significant).

Related to Figure 1.

Journal of Cell Science • Supplementary information

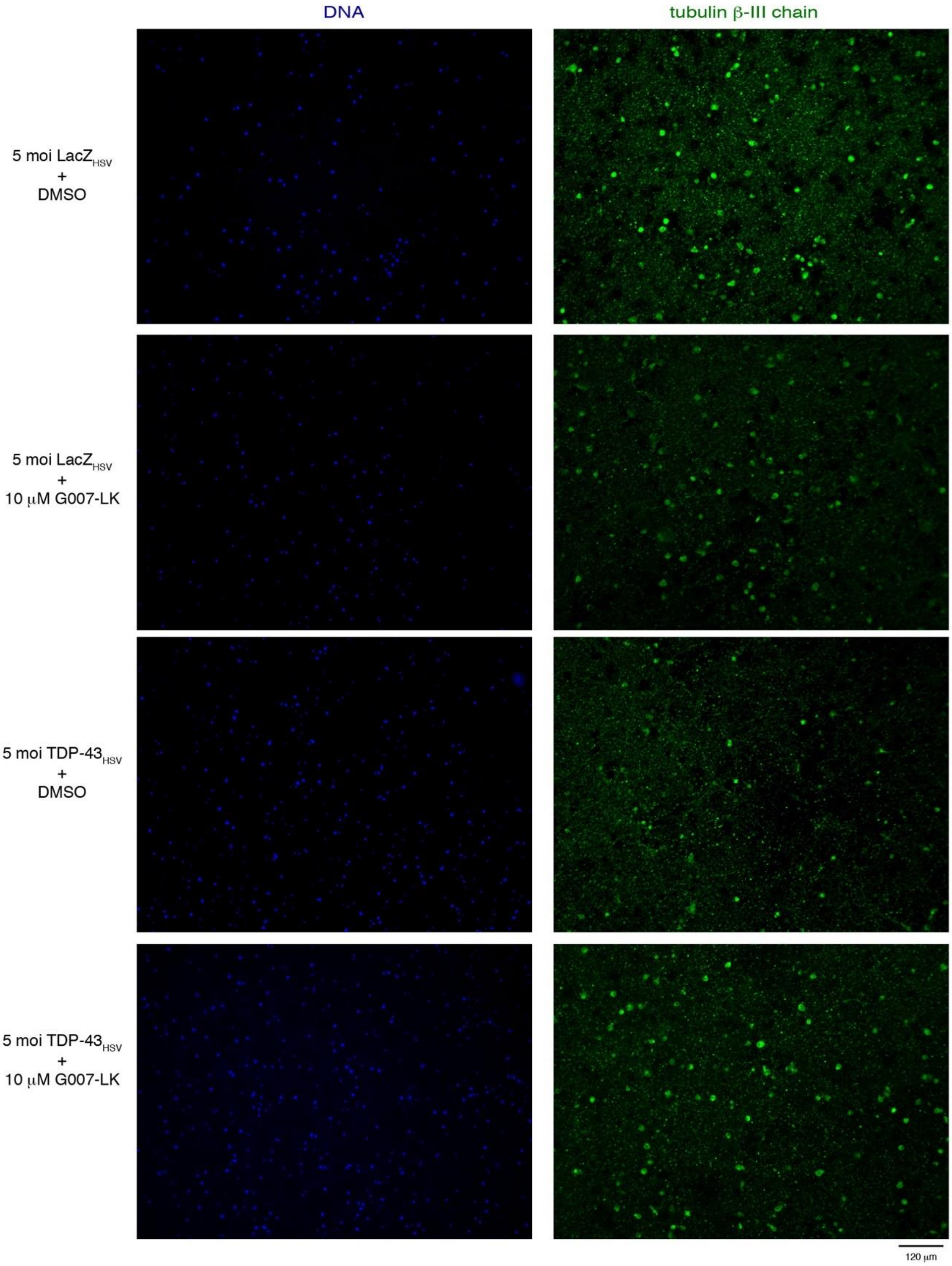

Journal of Cell Science • Supplementary information

Figure S2: Rat primary cortical neurons infected with TDP-43_{HSV} or $LacZ_{HSV}$ and treated with either vehicle control or the Tnks-1/2 inhibitors G007-LK.

Cortical neurons isolated from Sprague Dawley embryos (E16-E18) were virally infected with either HSV-LacZ or HSV-TDP-43 and treated with DMSO or G007-LK after 15-18 days in vitro (DIV). 7d post infection (DPI), neurons were fixed and immunostained with the neuronal marker tubulin β-III chain and counterstained with Hoechst. These are the same images presented in Fig. 7 here the images have been expanded and the Hoechst and tubulin β-III chain signals have been separated.

Related to Figure 1**.**

Journal of Cell Science • Supplementary information

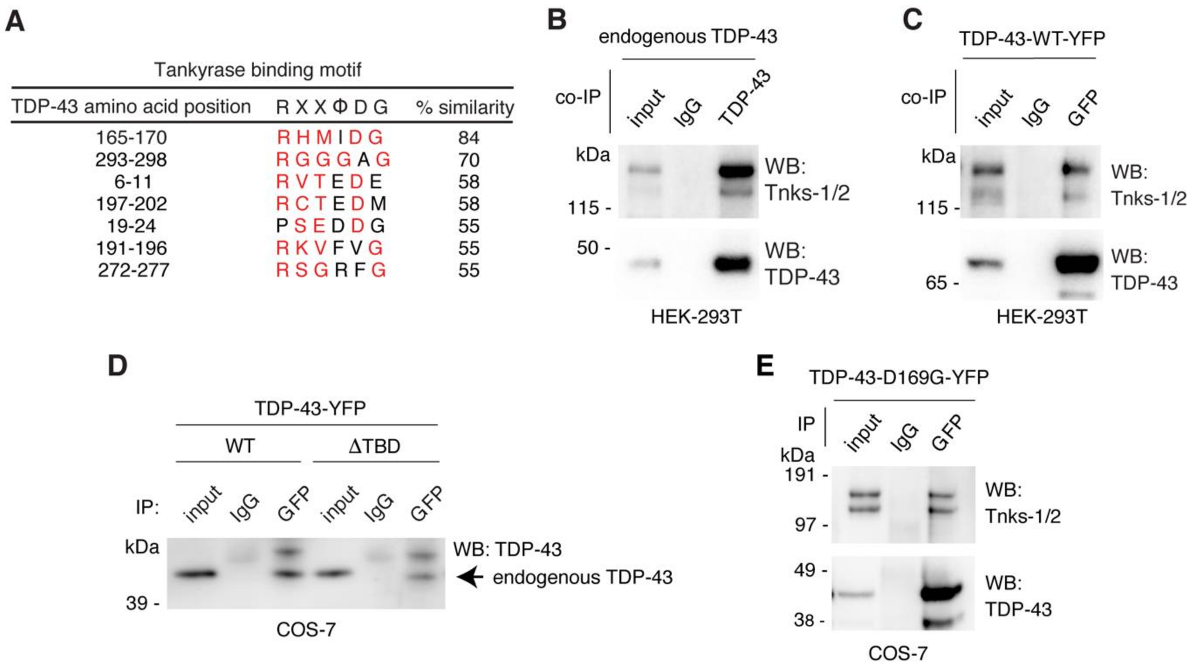

Figure S3. TDP-43 co-immunoprecipitates with tankyrase-1/2.

A. Endogenous Tnks-1/2 co-immunoprecipitated with endogenous TDP-43 in HEK-293T cells.

B. Endogenous Tnks-1/2 co-immunoprecipitated with TDP-43-WT-YFP expressed in HEK-293T cells.

C. The consensus of the Tankyrase-binding motif (TBD), RxxΦDG (where x represents any amino acid and Φ is a small hydrophobic amino acid) (Guettler et al., 2011; Sbodio and Chi, 2002), was aligned to TDP-43 using the PATTINPROT search engine (Combet et al., 2000). Table lists all regions with sequence similarity to the TBD identified in TDP-43. The region with highest amino acid identity to the TBM (amino acids 165-170) was mutated in this study.

D. TDP-43-WT-YFP and TDP-43-ΔTBD-YFP both co-immunoprecipitated with endogenous TDP-43 in COS-7 cells.

E. Endogenous Tnks-1/2 co-immunoprecipitated with TDP-43-D169G-YFP expressed in COS-7 cells.

Related to Figure 2.

Mean Levels of TDP-43-YFP upon cycloheximide treatment

	WT		ΔTBD		R165A		H166A		M167A		I168A		D169A		G170A	
Time (h)	RI	SD	RI	SD	RI	SD	RI	SD	RI	SD	RI	SD	RI	SD	RI	SD
0	1.00	0.00	1.00	0.00	1.00	0.00	1.00	0.00	1.00	0.00	1.00	0.00	1.00	0.00	1	0
24	0.66	0.22	0.24	0.01	0.73	0.22	0.37	0.07	0.51	0.06	0.27	0.03	0.66	0.20	0.7	0.14
48	0.06	0.03	0.01	0.01	0.03	0.02	0.06	0.03	0.06	0.02	0.01	0.01	0.09	0.06	0.1	0.05

RI: relative intensity
SD: standard deviation

Figure S4: Mean levels of TDP-43-YFP

Cells expressing TDP-43-YFP were treated with cycloheximide and TDP-43-YFP levels were measured by immunoblot. The table presents the mean and standard deviation from 3 independent experimental repeats.

Related to Figure 3.

Journal of Cell Science • Supplementary information

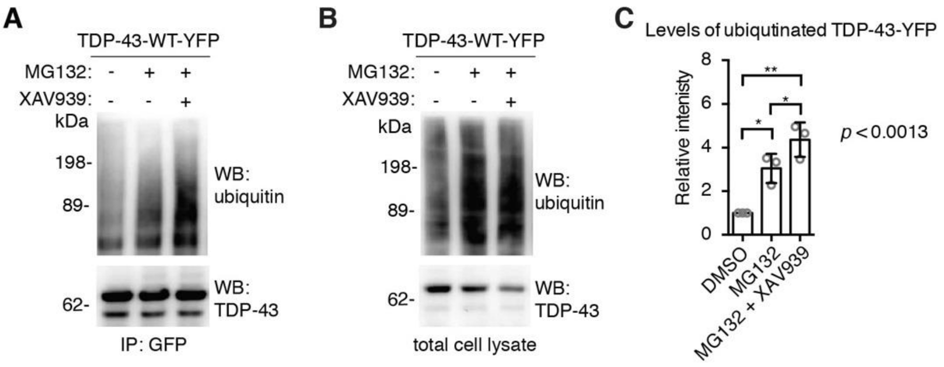

Figure S5: Treatment with XAV939 enhances MG132-induced ubiquitination of TDP-43-YFP.

A-B: Cells expressing TDP-43-WT-YFP were exposed to vehicle (DMSO), MG132 alone, or MG132 and the Tnks-1/2 inhibitor XAV939 (1 μM). (A) Immunoprecipitated TDP-43-YFP and (B) total cell lysates immunoblotted for ubiquitin and TDP-43.

C: Co-treatment with XAV939 and MG132 significantly increased the levels of ubiquitinated TDP-43-YFP compared to MG132 alone. Mean ±s.d. of 3 independent experiments. One-way ANOVA (where P=0.0013) and a Holm-Sidak's test were used to reveal pairwise significance (* P< 0.05, ** P<0.01).

Related to Figure 4.

Journal of Cell Science • Supplementary information

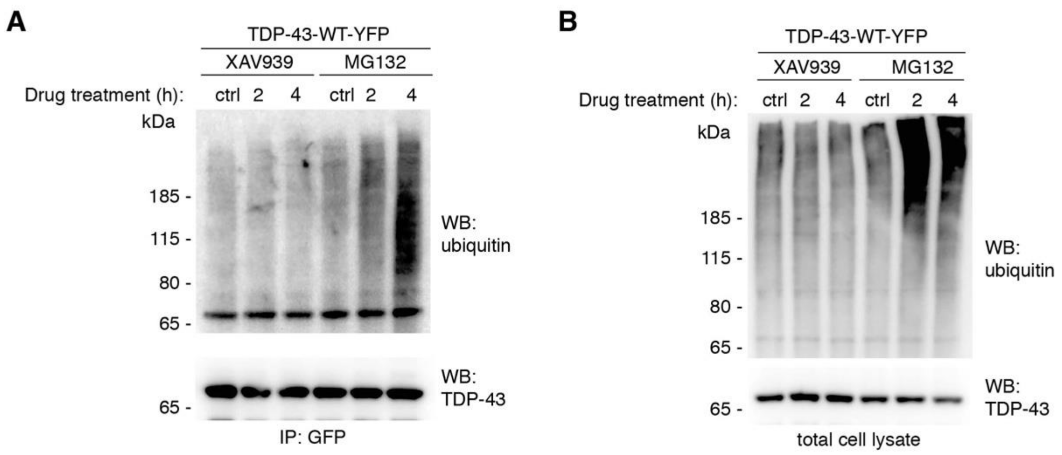

Figure S6: Treatment with XAV939 alone does not lead to ubiquitination of TDP-43.

A-B. Cells expressing TDP-43-WT-YFP were exposed to vehicle (DMSO), 1 µM XAV939 or MG132 10 µM. (A) Immunoprecipitated TDP-43-YFP and (B) total cell lysate immunoblotted for ubiquitin and TDP-43. Treatment with XAV939 alone did not lead to the ubiquitination of TDP-43; treatment with MG132 increased ubiquitination of TDP-43.

Related to Figure 4.

Journal of Cell Science • Supplementary information

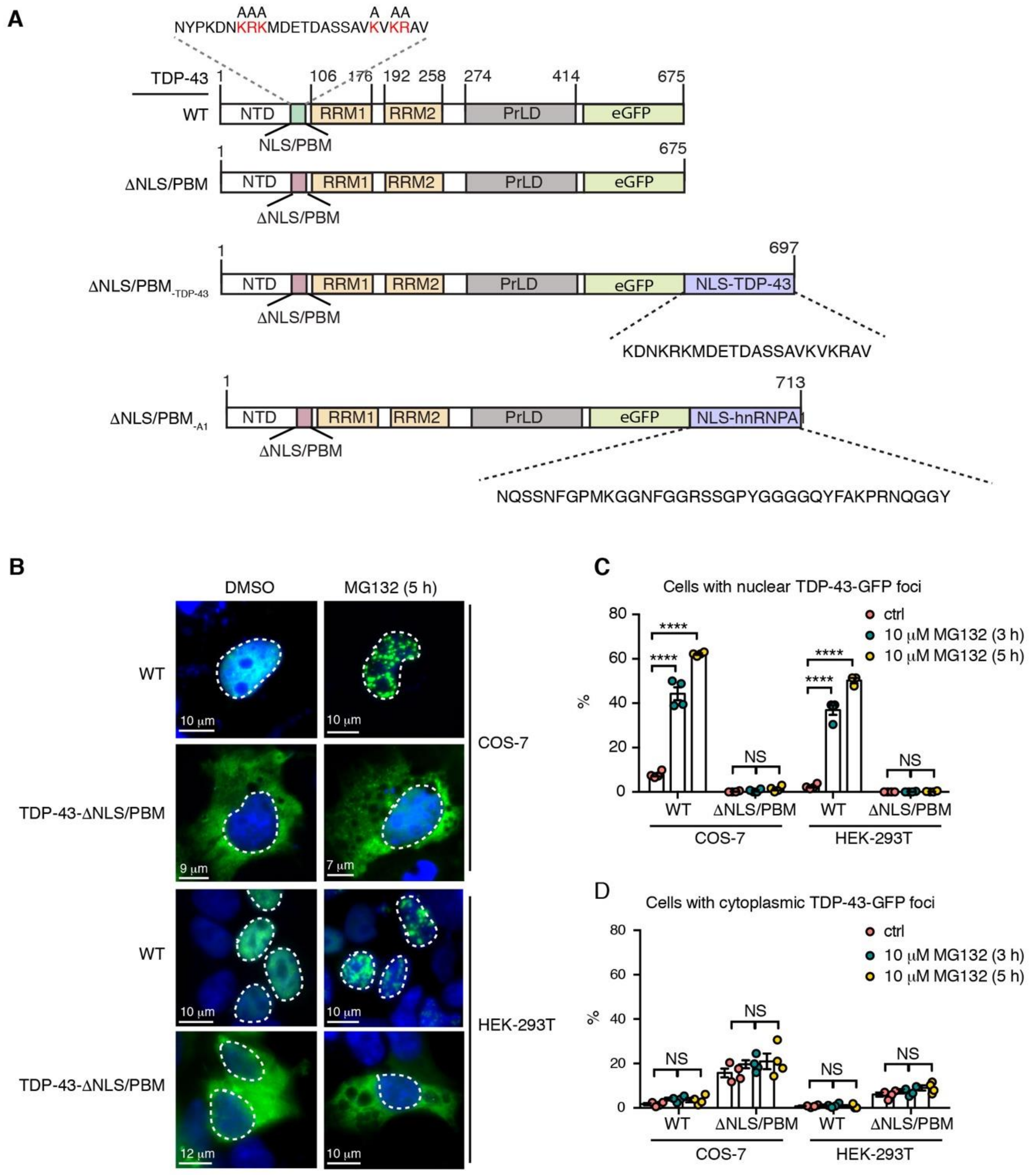

Figure S7. Treatment with MG132 leads to selective accumulation of TDP-43 in the nucleus of both COS-7 cells and HEK-293T cells.

A. The NLS from TDP-43 (TDP-43-ΔPBM$_{TDP-43}$) and the NLS from hnRNPA1 (TDP-43-ΔPBM$_{A1}$) was inserted immediately upstream of the stop codon of TDP-43-ΔNLS/PBM. The encoded amino acids inserted into each plasmid construct are indicated. TDP-43 domains: NTD, N-terminal domain (amino acids 1-80); NLS, nuclear localization sequence; PBM: PAR-binding motif; RRM, RNA recognition motif; PrLD: prion-like domain; the amino acids in red in the NLS/PBM were mutated to alanine, this mutation (ΔPBM/NLS) inhibits nuclear import of TDP-43.

Journal of Cell Science • Supplementary information

B. COS-7 and HEK-293T cells expressing TDP-43-WT-GFP or TDP-43-ΔPBM-GFP were exposed to a control (DMSO) or 10 μM MG132 for 5 h. TDP-43-WT-GFP formed nuclear foci upon MG132 treatment, while TDP-43-ΔPBM remained diffuse in the cytoplasm. Cells were fixed and counterstained with Hoechst.

C. In both COS-7 cells and HEK-293T cells, treatment with 10 μM MG132 for 3 h and 5 h led to a significant increase in the percentage of cells with nuclear foci of TDP-43-WT-GFP. Mean±s.e.m. of 3 independent experiments. Two-way ANOVA and a Tukey's test were used to reveal pairwise significance (*****P*<0.0001, NS: not significant).

D. In both COS-7 cells and HEK-293T cells, treatment with 10 μM MG132 for 3 h and 5 h did not lead TDP-43-GFP foci in the cytoplasm. Mean±s.e.m. of 3 independent experiments. Two-way ANOVA and a Tukey's test were used to reveal pairwise significance (NS: not significant).

Related to Figure 6.

Journal of Cell Science • Supplementary information

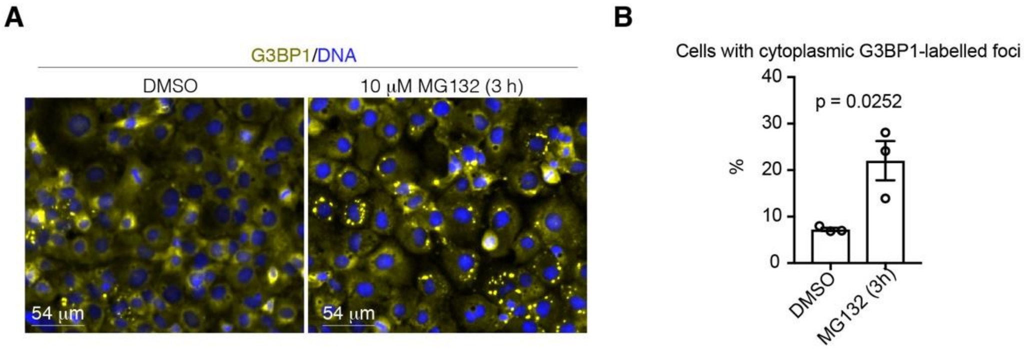

Figure S8: MG132 leads to accumulation of G3BP1-labelled foci in the cytoplasm.

A. COS-7 cells treated with vehicle (DMSO) or 10 µM MG132 were fixed and immunolabeled with G3BP1 (yellow) and counterstained with Hoechst (blue).

B. The percentage of cells with G3BP1-labelled foci in the cytoplasm was calculated. MG132 (10 µM) for 3 h lead to a significant increase in G3BP1-labelled foci in the cytoplasm. The mean±s.e.m. of 3 independent experiments is presented. An unpaired t test was used. These data are consistent with previous data that demonstrate that G3BP1-labeled stress granules form after 3 h of treatment with 10 µM MG132 (Mazroui et al., 2007).

Related to Figure 6.

Supplemental references

Combet, C., C. Blanchet, C. Geourjon, and G. Deleage. 2000. NPS@: network protein sequence analysis. *Trends Biochem Sci*. 25:147-150.

Guettler, S., J. LaRose, E. Petsalaki, G. Gish, A. Scotter, T. Pawson, R. Rottapel, and F. Sicheri. 2011. Structural basis and sequence rules for substrate recognition by Tankyrase explain the basis for cherubism disease. *Cell*. 147:1340-1354.

Mazroui, R., S. Di Marco, R.J. Kaufman, and I.E. Gallouzi. 2007. Inhibition of the ubiquitin-proteasome system induces stress granule formation. *Molecular biology of the cell*. 18:2603-2618.

Sbodio, J.I., and N.W. Chi. 2002. Identification of a tankyrase-binding motif shared by IRAP, TAB182, and human TRF1 but not mouse TRF1. NuMA contains this RXXPDG motif and is a novel tankyrase partner. *The Journal of biological chemistry*. 277:31887-31892.
