# Supplementary material for: TDP-43, a protein central to amyotrophic lateral sclerosis, is destabilized by tankyrase-1 and -2
Source: J Cell Sci. 2020 Jun 23;133(12):jcs245811. doi: 10.1242/jcs.245811 (PMC7328137; doi:10.1242/jcs.245811)
Supplement: Reviewer comments [file joces-133-245811_review_history.pdf]

Journal of
Cell Science

TDP-43, a protein central to amyotrophic lateral sclerosis, is destabilized by tankyrase-1 and -2

Leeanne McGurk, Olivia M. Rifai and Nancy M. Bonini
DOI: 10.1242/jcs.245811

Editor: Giampietro Schiavo

Review timeline

Original submission:	29 February 2020
Editorial decision:	1 April 2020
First revision received:	17 April 2020
Accepted:	24 April 2020

Original submission

First decision letter

MS ID#: JOCES/2020/245811

MS TITLE: TDP-43 a protein central to amyotrophic lateral sclerosis is destabilized by tankyrase-1 and -2

AUTHORS: Leeanne McGurk, Olivia Rifai, and Nancy Bonini
ARTICLE TYPE: Research Article

We have now reached a decision on the above manuscript.

To see the reviewers' reports and a copy of this decision letter, please go to: https://submit-jcs.biologists.org and click on the 'Manuscripts with Decisions' queue in the Author Area. (Corresponding author only has access to reviews.)

As you will see, the reviewers viewed enthusiastically your work and returned favourable reports but raised few critical points that will require amendments to your manuscript. I hope that you will be able to carry these out, because I would like to be able to accept your paper.

We are aware that you may currently be unable to access the lab to undertake experimental revisions. If it would be helpful, we encourage you to contact us to discuss your revision in greater detail. Please send us a point-by-point response indicating where you are able to address concerns raised (either experimentally or by changes to the text) and where you will not be able to do so within the normal timeframe of a revision. We will then provide further guidance. Please also note that we are happy to extend revision timeframes as necessary.

Please ensure that you clearly highlight all changes made in the revised manuscript. Please avoid using 'Tracked changes' in Word files as these are lost in PDF conversion.

I should be grateful if you would also provide a point-by-point response detailing how you have dealt with the points raised by the reviewers in the 'Response to Reviewers' box. Please attend to all of the reviewers' comments. If you do not agree with any of their criticisms or suggestions please explain clearly why this is so.

© 2020. Published by The Company of Biologists under the terms of the Creative Commons Attribution License (https://creativecommons.org/licenses/by/4.0/).

Reviewer 1

Advance summary and potential significance to field

McGurk et al. continue their seminal work on functional TDP-43 interactions with PARPs. They establish that TDP-43 binds to tankyrases via a newly defined domain. Interestingly, such interaction stabilized TDP-43, evidently by removing the protein from nuclear proteasomal degradation. Conversely, blocking TNKS-1/2 reduced TDP-43 levels and toxicity in primary rat cortical neurons. Overall the work is important, well done and the results are of high quality.

Comments for the author

(1) Thoroughly proofread the manuscript text. There are many typos and mistakes, including:
a. Page 5, line 18: "…TBD and the RNA-binding regions are on opposite sides of RRM1 (Fig. 1I not J)."
b. Page 5, line 23: "…amino acids (…) on the external surface of the β-strand (M167 delete A)…"
c. Page 5, line 38: "steady-state levels" mean the sum of protein synthesis and degradation, as determined in straight Western blots. What is measured in the CHX chase experiments is the rate of degradation. Use proper wording throughout the description of Figure 2.
d. The title of "Figure 2: TNKS-1/2 does not promote degradation of TDP-43" is somewhat misleading. Better something like "TNKS-1/2 regulate TDP-43 degradation". Same for Figure 3.
e. Page 6, line 1: Fig. 2 delete A-F as it refers to the whole Figure 2.
f. Page 6, line 17: "turnover" refers to the sum of degradation and synthesis – specifically state "proteasomal degradation".
g. Page 6, last line: correct Fig. S7 not S5.
h. Page 7, last sentence: delete (Fig. 6 D) - there is no such figure.
i. Legend to Fig. 7A: "…infected with (…) HSV-TDP-43…" not TDP-4.
j. Page 8: refer to Fig. S9 at the appropriate place.
k. Page 10, line 32: hexanucleotide not hexucleotide
l. Consistently spell C9ORF72.

(2) Enlarge the amino acid labelings in Fig. 1J, they are too small to read.

(3) It is funny that the most conserved residue D169 in the putative TBP appears to play no role at all, as it is also conspicuously D169G mutated and disease-linked.

(4) As mentioned above, the wording for Figure 2 should be more precise. And although the data do support the conclusions about TDP-43 breakdown, ideally degradation rates would be measured in metabolic pulse-chase experiments. The translation inhibitor cycloheximide has a remote chance of affecting the RNA-binding and stress granule-associated protein TDP-43. At least decay rates with sufficient numbers of time points after CHX block and appropriate curve fitting to calculate half-life times would be nice.

(5) Delete Fig. 3G the concept is clear enough at that point and redundant with Fig. 8.

(6) Most key experiments were done with YFP-tagged TDP-43. It is possible that this large protein portion affects ubiquitinylation and aggregation. Confirm with untagged or where feasible for the endogenous TDP-43 protein.

(7) As was brought up by the previous reviewers we also wonder about the molecular mechanisms targeting TDP-43 for proteasomal degradation in the nucleus and cytoplasm, respectively. Could this reflect specific PARylation sites and effects? It would be informative to check the PARylation states in detail under these conditions.

© 2020. Published by The Company of Biologists under the terms of the Creative Commons Attribution License (https://creativecommons.org/licenses/by/4.0/).

Reviewer 2

Advance summary and potential significance to field

McGurk and colleagues show that Tankyrase-1/2 (Tnks-1/2) stabilizes TDP-43 in the cytoplasm, which subsequently leads to a reduced turnover of TDP-43 by nuclear proteasomes. The authors previously observed that Tnks-1/2 inhibitors lead to reduced TDP-43 induced neurotoxicity in Drosophila. Here they follow up on the underlying cellular mechanism.
The first experiment in the manuscript demonstrates by co-immunoprecipitation that Tnks-1/2 binds to TDP-43. The authors also define a Tankyrase-binding motif (TBM) in the TDP-43 sequence. They demonstrate further that the TBM regulates TDP-43 turnover in CHX shut-off experiments. Wt TDP-43 is degraded faster than ΔTBM TDP-43. Also, inhibition of Tnks-1/2 with a specific inhibitor leads to faster degradation. Using this inhibitor provides a clean way to show that indeed the interaction between Tnks-1/2 and TDP-43 is crucial for stabilization of TDP-43 in the cytoplasm. Regarding the ΔTBM TDP-43 mutant, I am missing an activity assay showing that TDP-43, besides lacking the interaction with Tnks-1/2, is still functional and correctly folded. This concern becomes even more important when McGurk at al. examine the ubiquitination of TDP-43 during MG132 treatment. They observe that ubiquitination is elevated when Tnks-1/2 is inhibited or TDP-43 is lacking the TBM. Observing an elevated ubiquitination of ΔTBM TDP-43 could also be explained by the formation of misfolded protein.
Next, the authors stain cells expressing wt TDP-43 as well as ΔTBM TDP-43 after treatment with MG132. They observe ubiquitin-positive foci in the nucleus that are co-localizing with TDP-43. However, the foci in the ΔTBM TDP-43 mutant seem to be larger. The authors explain this with the elevated ubiquitination of TDP-43 observed by western blot. Co-localization should also be examined with a K48 specific antibody.
TDP-43 is completely localized to the nucleus upon Tnks-1/2 inhibition, in support of the conclusion that Tnks-1/2 must stabilize TDP-43 in the cytoplasm which leads to reduced import into the nucleus, reduced ubiquitination and subsequently reduced proteasomal degradation.
In a final experiment the authors extend an observation previously made in Drosophila neurons: Upon treatment with Tnks-1/2 inhibitor, TDP-43 induced neurotoxicity is reduced in murine primary neurons. It might be a good idea to start the paper with this experiment, helping the reader to connect the present study with previous observations.
Overall this is an interesting, well performed study. Publication is recommended after minor revisions as outlined.

Comments for the author

See above.

First revision

Author response to reviewers' comments

Please find the Reviewers comments in black and our response in Red:

We thank the Reviewers for their thoughtful comments and provide extensive new data to address their concerns.

Reviewer 1 Comments for the Author:

(1) Thoroughly proofread the manuscript text. There are many typos and mistakes, including:
a. Page 5, line 18: "…TBD and the RNA-binding regions are on opposite sides of RRM1 (Fig. 1I not J)."
b. Page 5, line 23: "…amino acids (…) on the external surface of the ß-strand (M167 delete A)…"

© 2020. Published by The Company of Biologists under the terms of the Creative Commons Attribution License (https://creativecommons.org/licenses/by/4.0/).

We thank the Reviewer for highlighting these errors, we have corrected them and have thoroughly proofread the manuscript.

c. Page 5, line 38: "steady-state levels" mean the sum of protein synthesis and degradation, as determined in straight Western blots. What is measured in the CHX chase experiments is the rate of degradation. Use proper wording throughout the description of Figure 2.

We thank the Reviewer for these comments. In the process of revising the manuscript Figure 2 has been relabelled Figure 3 in the final edited manuscript.

Page 6, line 18 now reads: "we treated cells with the protein-synthesis inhibitor cycloheximide and measured the degradation of endogenous TDP-43 in the presence of vehicle (DMSO) or the Tnks-1/2 inhibitor XAV939."

Page 6, line 22 now reads: "This finding indicates that inhibition of Tnks-1/2 promotes the degradation of endogenous TDP-43."

Page 6, line 23 now reads: "To rule out potential off-target effects of the Tnks-1/2 inhibitor, we compared the degradation of TDP-43-WT to the forms of TDP-43 unable to interact with Tnks-1/2 (TDP-43-ΔTBD, -H166A and -I168A)."

Page 6, line 29 now reads: "Together, these data indicate that loss of the Tnks-1/2 interaction leads to increased degradation of TDP-43 and suggest that under normal conditions Tnks-1/2 functions to stabilize TDP-43."

Page 10, line 23 now reads: "Our data further suggest that turnover of TDP-43 by the nuclear proteasome is important for regulating TDP-43 degradation."

d. The title of "Figure 2: TNKS-1/2 does not promote degradation of TDP-43" is somewhat misleading. Better something like "TNKS-1/2 regulate TDP-43 degradation". Same for Figure 3.

We thank the Reviewer for this suggestion, we have edited the Figure legends accordingly. These Figures have been re-labelled from Figure 2 and 3 in the original manuscript to Figures 3 and 4 in the revised manuscript.

Figure 3. Tnks-1/2 regulates the degradation of TDP-43.
Figure 4: Tnks-1/2 regulates the ubiquitination of TDP-43.

e. Page 6, line 1: Fig. 2 delete A-F as it refers to the whole Figure 2.

We have edited the manuscript accordingly.

f. Page 6, line 17: "turnover" refers to the sum of degradation and synthesis – specifically state "proteasomal degradation".

Page 7, line 2 now reads:" MG132-induced ubiquitination of TDP-43 and increased proteasomal degradation of the protein.

g. Page 6, last line: correct Fig. S7 not S5.

We have edited the manuscript accordingly.

h. Page 7, last sentence: delete (Fig. 6 D) - there is no such figure.

We have corrected this error it now references Fig.8

i. Legend to Fig. 7A: "…infected with (…) HSV-TDP-43…" not TDP-4
j. Page 8: refer to Fig. S9 at the appropriate place.
k. Page 10, line 32: hexanucleotide not hexucleotide

© 2020. Published by The Company of Biologists under the terms of the Creative Commons Attribution License (https://creativecommons.org/licenses/by/4.0/).

We have edited the manuscript accordingly.

l. Consistently spell C9ORF72.

We apologize and we have corrected to the official gene name *C9orf72* and protein name C9orf72. This terminology follows the uniprot guidelines at https://www.uniprot.org/uniprot/Q96LT7

(2) Enlarge the amino acid labelings in Fig. 1J, they are too small to read.

Thank you for pointing this out. We have edited the Figure accordingly.

(3) It is funny that the most conserved residue D169 in the putative TBP appears to play no role at all, as it is also conspicuously D169G mutated and disease-linked.

We agree with the Reviewer that it is perplexing that the D169 position when mutated to glycine or alanine does not alter the coimmunoprecipitation of TDP-43 with Tnks-1/2.

(4) As mentioned above, the wording for Figure 2 should be more precise. And although the data do support the conclusions about TDP-43 breakdown, ideally degradation rates would be measured in metabolic pulse-chase experiments. The translation inhibitor cycloheximide has a remote chance of affecting the RNA-binding and stress granule-associated protein TDP-43. At least decay rates with sufficient numbers of time points after CHX block and appropriate curve fitting to calculate half-life times would be nice.

We thank the Reviewer for their comments. We have amended this (now Figure 3) and corrected the language to be more precise on this point throughout the text.

(5) Delete Fig. 3G the concept is clear enough at that point and redundant with Fig. 8

Thank you for noting this. We have now deleted Figure panel 3G.

(6) Most key experiments were done with YFP-tagged TDP-43. It is possible that this large protein portion affects ubiquitinylation and aggregation. Confirm with untagged or where feasible for the endogenous TDP-43 protein.

We thank the Reviewer for pointing this out. We have data included that demonstrates that endogenous TDP-43 is degraded more rapidly in the presence of the Tnks-1/2 inhibitor XAV939 in Figures 2A and B. This finding is consistent with what we observed with TDP-43-YFP with mutations that prevent the co-immunoprecipitation with Tnks- 1/2 vs the wild-type form of TDP-43. Collectively, these data indicate that endogenous TDP-43 and the tagged form of TDP-43 (TDP-43-YFP) behave similarly. We have edited the manuscript to make it more clear that the data on endogenous TDP-43 is consistent with the YFP tagged forms as follows:

Page 6, line 25 now reads: "Consistent with the inhibitor data on endogenous TDP- 43 (Fig. 2, A and B), the levels of TDP-43 unable to interact with Tnks-1/2 (TDP-43-ΔTBD, -H166A and -I168A) were significantly reduced ($p < 0.0009$) compared to TDP-43 that interacts with Tnks-1/2 (TDP-43-WT, -R165A, -M167A, -D169A and - G170A) (Fig. 2, E-F and Fig. S2)."

(7) As was brought up by the previous reviewers we also wonder about the molecular mechanisms targeting TDP-43 for proteasomal degradation in the nucleus and cytoplasm, respectively. Could this reflect specific PARylation sites and effects? It would be informative to check the PARylation states in detail under these conditions.

We agree with the Reviewer that the mechanism underlying the accumulation of TDP-43 in the nucleus vs the cytoplasm and how Tnks1/2 relates to this is intriguing. We have checked PARylation status of TDP-43 and have not yet observed TDP-43 to be directly modified under any of the conditions that we have tested (also McGurk et al. Molecular Cell). Further insight into this important question will be the focus of future studies.

© 2020. Published by The Company of Biologists under the terms of the Creative Commons Attribution License (https://creativecommons.org/licenses/by/4.0/).

Reviewer 2 Advance Summary and Potential Significance to Field:
McGurk and colleagues show that Tankyrase-1/2 (Tnks-1/2) stabilizes TDP-43 in the cytoplasm, which subsequently leads to a reduced turnover of TDP-43 by nuclear proteasomes. The authors previously observed that Tnks-1/2 inhibitors lead to reduced TDP- 43 induced neurotoxicity in Drosophila. Here they follow up on the underlying cellular mechanism.The first experiment in the manuscript demonstrates by co-immunoprecipitation that Tnks-1/2 binds to TDP-43. The authors also define a Tankyrase binding motif (TBM) in the TDP-43 sequence. They demonstrate further that the TBM regulates TDP-43 turnover in CHX shut-off experiments. Wt TDP-43 is degraded faster than ΔTBM TDP-43. Also, inhibition of Tnks-1/2 with a specific inhibitor leads to faster degradation. Using this inhibitor provides a clean way to show that indeed the interaction between Deletion of the TBD did not affect all interactions, as it had no effect on the capacity of TDP-43-ΔTBD to co-immunoprecipitate with endogenous TDP-43 (Fig. S1d).Tnks- 1/2 and TDP-43 is crucial for stabilization of TDP-43 in the cytoplasm.

Regarding the ΔTBM TDP-43 mutant, I am missing an activity assay showing that TDP-43, besides lacking the interaction with Tnks-1/2, is still functional and correctly folded. This concern becomes even more important when McGurk at al. examine the ubiquitination of TDP-43 during MG132 treatment. They observe that ubiquitination is elevated when Tnks-1/2 is inhibited or TDP-43 is lacking the TBM. Observing an elevated ubiquitination of ΔTBM TDP-43 could also be explained by the formation of misfolded protein.

We thank the Reviewer for this comment. We demonstrate that TDP-43-TBD still co-immunoprecipitates with endogenous TDP-43, indicating that the mutant protein maintains its ability to interact properly with the endogenous WT protein. We have added text to make this clear.

Page 5, Line 34 now reads: “Importantly, deletion of the TBD did not affect all interactions, as it had no effect on the capacity of TDP-43-ΔTBD to co- immunoprecipitate with endogenous TDP-43 from cellular lysates (Fig. S3 D).”

Next, the authors stain cells expressing wt TDP-43 as well as ΔTBM TDP-43 after treatment with MG132. They observe ubiquitin-positive foci in the nucleus that are co-localizing with TDP-43. However, the foci in the ΔTBM TDP-43 mutant seem to be larger. The authors explain this with the elevated ubiquitination of TDP-43 observed by western blot. Co-localization should also be examined with a K48 specific antibody.

We thank the Reviewer for this comment. While we agree more details on the ubiquitination of TDP-43 are of interest, we hope that you will agree that additional details and additional focused study of the ubiquitination of TDP-43 (for example, type of linkages, and type of linkage in the nucleus vs cytoplasm, and the impact of proteasome inhibition and Tankyase1/2 activity on that) are beyond the scope of this particular study.

TDP-43 is completely localized to the nucleus upon Tnks-1/2 inhibition, in support of the conclusion that Tnks-1/2 must stabilize TDP-43 in the cytoplasm, which leads to reduced import into the nucleus, reduced ubiquitination and subsequently reduced proteasomal degradation. In a final experiment the authors extend an observation previously made in Drosophila neurons: Upon treatment with Tnks-1/2 inhibitor, TDP-43 induced neurotoxicity is reduced in murine primary neurons. It might be a good idea to start the paper with this experiment, helping the reader to connect the present study with previous observations.

We thank the Reviewer for this helpful suggestion and we have amended the manuscript accordingly.

Overall this is an interesting, well performed study. Publication is recommended after minor revisions as outlined.

Reviewer 2 Comments for the Author:
See above.

FINAL COMMENTS:

© 2020. Published by The Company of Biologists under the terms of the Creative Commons Attribution License (https://creativecommons.org/licenses/by/4.0/).

We thank the Reviewers for their enthusiasm for our work, and for their extremely helpful comments that have improved the impact of our findings.

We hope that with these revisions, the Editor and Reviewers will now find the work acceptable for publication in *Journal of Cell Science*.

Second decision letter

MS ID#: JOCES/2020/245811

MS TITLE: TDP-43 a protein central to amyotrophic lateral sclerosis is destabilized by Tankyrase-1/2

AUTHORS: Leeanne McGurk, Olivia Rifai, and Nancy Bonini
ARTICLE TYPE: Research Article

I am happy to tell you that your manuscript has been accepted for publication in Journal of Cell Science, pending standard ethics checks.

© 2020. Published by The Company of Biologists under the terms of the Creative Commons Attribution License (https://creativecommons.org/licenses/by/4.0/).
